# Earth-Abundant W_18_O_49_ Coupled with Minimal Pt for Enhanced Hydrogen Evolution under Dark and Visible Light Conditions

**DOI:** 10.1021/acsami.4c22952

**Published:** 2025-03-11

**Authors:** Hugo L. S. Santos, Md Mofakkharulhashan, Shiqi Wang, Eric V. Formo, Mykhailo Chundak, Mikko Ritala, Wenyi Huo, Pedro H. C. Camargo

**Affiliations:** † Department of Chemistry, University of Helsinki, A.I. Virtasen Aukio 1, P.O. Box 55, Helsinki FIN-00560, Finland; ‡ Georgia Electron Microscopy, University of Georgia, Athens, Georgia 30602, United States; § College of Mechanical and Electrical Engineering, Nanjing Forestry University. Nanjing 210037, P. R. China; ∥ NOMATEN Centre of Excellence, National Centre for Nuclear Research. Otwock 05-400, Poland

**Keywords:** plasmonic catalysis, hydrogen evolution reaction, W_18_O_49_, platinum catalysts, ultralow loadings, earth-abundant materials

## Abstract

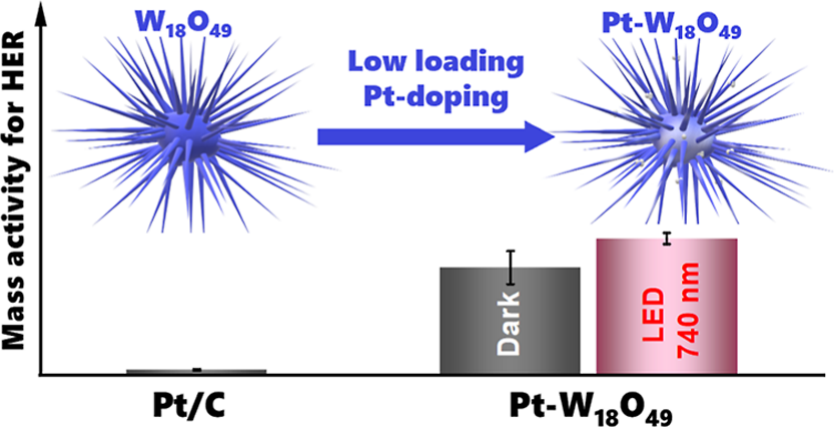

The development of cost-effective and efficient electrocatalysts for the hydrogen evolution reaction (HER) is critical to advancing green hydrogen production technologies. Here, we present a plasmonic tungsten oxide (W_18_O_49_) material integrated with ultralow platinum (Pt) loadings (0.4, 0.8, and 1.6 wt %) that delivers high HER performances under both dark and visible light conditions. The 0.4 wt % Pt–W_18_O_49_ catalyst exhibits remarkable mass activity, outperforming commercial Pt/C by factors of 15 and 30 under dark and 740 nm LED illumination, respectively. Density functional theory (DFT) calculations reveal that the synergy between Pt and plasmonically active W_18_O_49_ optimizes charge transfer and hydrogen adsorption, resulting in lowered energy barriers for HER kinetics. Furthermore, plasmonic excitation of W_18_O_49_ enhances catalytic activity by facilitating electron transfer. This work introduces a scalable, cost-effective strategy for combining earth-abundant plasmonic materials with minimal Pt usage, providing a pathway toward high-efficiency HER catalysts. These findings highlight the potential of plasmonic-catalyst integration in green hydrogen technologies.

## Introduction

Green hydrogen has emerged as a key solution in transitioning toward a sustainable energy economy.^1−3^ However, its widespread adoption relies on overcoming efficiency challenges in the hydrogen evolution reaction (HER) and oxygen evolution reaction (OER), both critical components of electrochemical water splitting.^4,5^ Platinum (Pt), a noble metal, remains the benchmark electrocatalyst for HER due to its exceptional activity and stability in acidic environments.^6,7^ Yet, the high cost and limited availability of Pt may pose significant barriers to its large-scale application.^8,9^

To address these challenges, significant research efforts have focused on reducing Pt loading or replacing it with earth-abundant alternatives, without sacrificing catalytic performance. Strategies such as single-atom catalysts, core–shell architectures, and alloying have demonstrated progress in optimizing Pt utilization.^10−12^ Additionally, integrating Pt with support materials, particularly transition metal oxides (TMOs), has proven effective in enhancing mass activity while reducing Pt content.^13,14^ However, many TMOs face limitations, including poor electrical conductivity and a scarcity of active sites for HER, which restrict their practical application.^13,15^

Among TMOs, mixed-valence tungsten oxide (W_18_O_49_) has emerged as a promising support material due to its distinctive structural and electronic properties.^16−19^ Unlike stoichiometric WO_3_, W_18_O_49_ is characterized by a high density of oxygen vacancies (V_o_), which confer metallic-like conductivity. Furthermore, W_18_O_49_ exhibits localized surface plasmon resonance (LSPR), enabling strong absorption in the visible-to-near-infrared (NIR) range, akin to conventional plasmonic metals such as gold and silver.^20,21^ This LSPR effect generates hot carriers that can enhance catalytic activity at adjacent sites, including Pt. Despite these promising attributes, the potential of W_18_O_49_ as a plasmonic support for ultralow-loading Pt HER catalysts remains underexplored.

In this work, we present a simple one-step solvothermal synthesis of Pt-modified W_18_O_49_ electrocatalysts with ultralow Pt loadings (0.4, 0.8, and 1.6 wt %). The resulting urchin-like W_18_O_49_ particles exhibited exceptional HER activity, surpassing commercial Pt/C under both dark and visible light conditions. Experimental analyses combined with density functional theory (DFT) calculations revealed that the synergistic interaction between Pt and W_18_O_49_ optimizes electronic structure, facilitating efficient charge transfer and improved hydrogen adsorption/desorption kinetics. DFT studies further confirmed that Pt at the Pt–W_18_O_49_ interface exhibits lower hydrogen adsorption energy barriers compared to Pt(111), driving the superior catalytic performance. These findings underscore the potential of Pt-modified W_18_O_49_ as a cost-effective and light-responsive HER catalyst, offering valuable insights for the design of advanced plasmon-enhanced electrocatalysts for sustainable hydrogen production.

## Result and Discussion

**Scheme 1:**
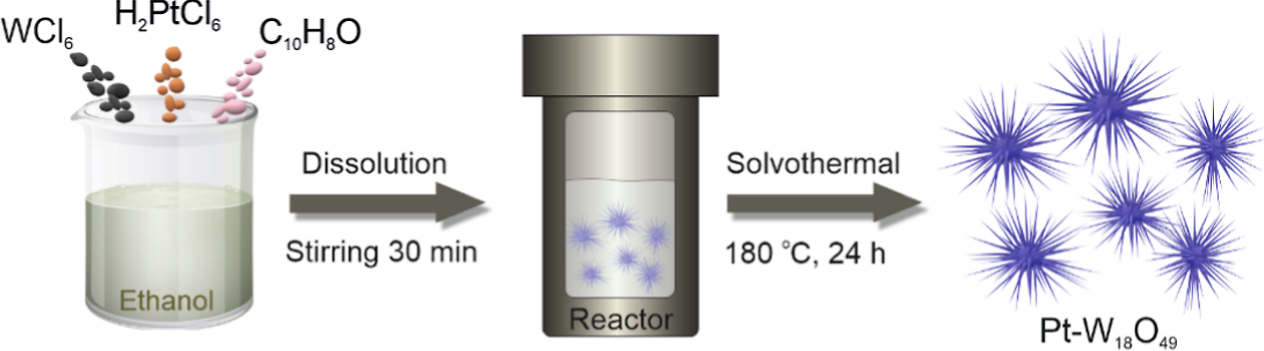
Pt–W_18_O_49_ Synthesis Schematic illustration of the Pt–W_18_O_49_ synthesis process.

Pt-modified W_18_O_49_ nanourchins were synthesized through a one step solvothermal method with ethanol as the solvent and 1-naphthol as a reducing agent, as presented in Scheme 1. No postsynthesis annealing was applied for the samples. They were washed, dried at 50 °C, and directly employed in HER catalysis. To achieve ultralow Pt loadings (<2 wt %), we systematically introduced 0.66 mg, 1.32 mg, or 2.64 mg of H_2_PtCl_6_·*x*H_2_O along with 100 mg of WCl_6_ in the synthesis to investigate how the composition under ultralow Pt loadings in Pt–W_18_O_49_ nanourchins affects HER activity. The resulting Pt contents, quantified by MP-AES, were 0.4, 0.8, and 1.6 wt % (Table S1), closely matching the theoretical values (0.43, 0.86, and 1.71 wt %, respectively), demonstrating precise control over Pt loading. These samples were accordingly labeled as 0.4Pt–W_18_O_49_, 0.8Pt–W_18_O_49_, and 1.6Pt–W_18_O_49_, respectively.

**Figure 1:**
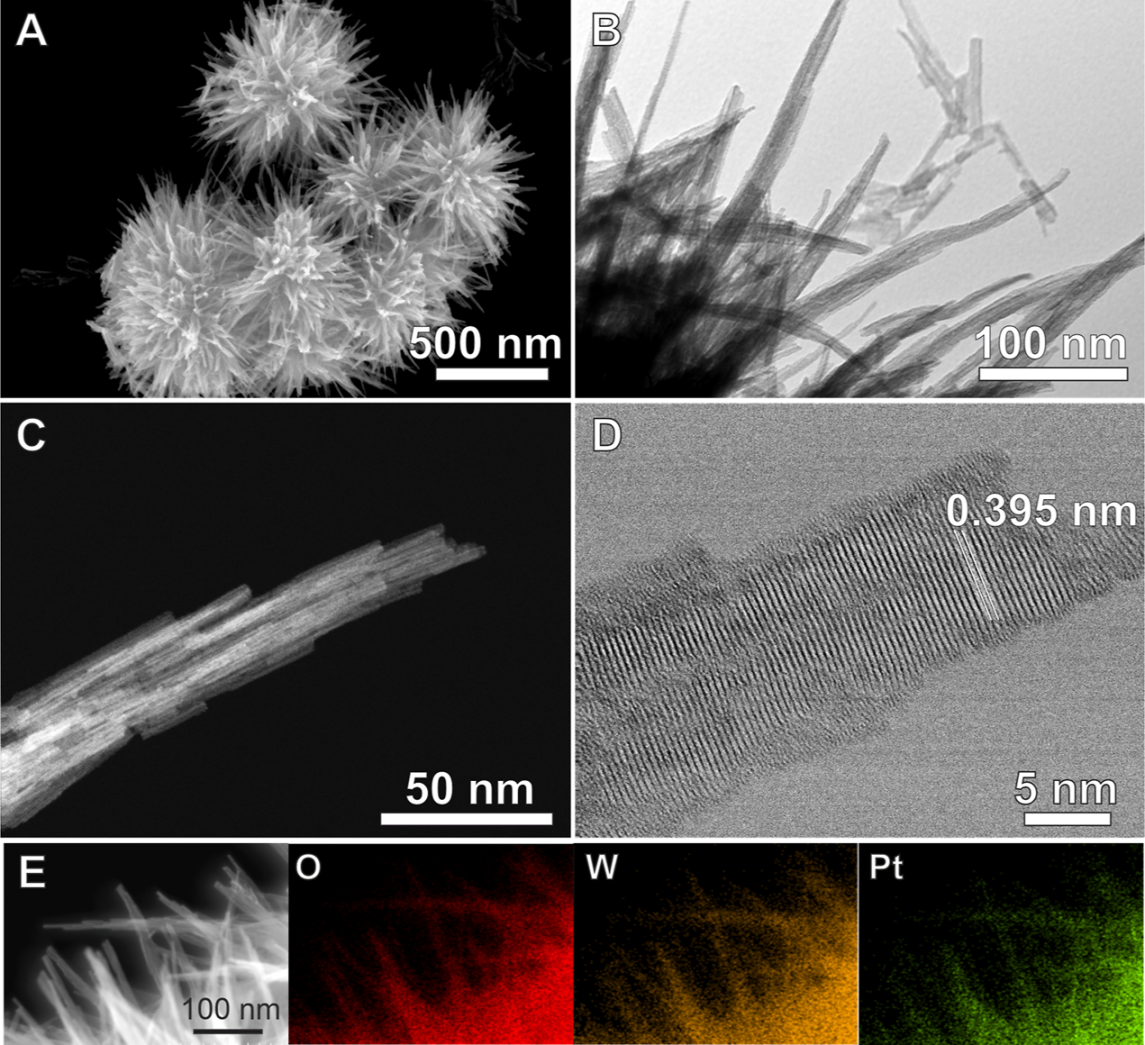
Structural and compositional characterization of 1.6Pt–W_18_O_49_ nanourchins. (A) SEM images depicting the nanourchin morphology of 1.6Pt–W_18_O_49._ (B) TEM image highlighting the bundle-like structure of the 1.6Pt–W_18_O_49_ nanourchins containing nanowires at their surface. (C) STEM-HAADF image showing the detailed morphology of individual nanowires in the 1.6Pt–W_18_O_49_ nanourchins. (D) HRTEM image showing the crystalline lattice fringes with an interplanar spacing of 0.395 nm, indicative of the W_18_O_49_ phase. (E) STEM-HAADF and corresponding STEM-EDS maps for the distribution of O (red), W (orange) and Pt (green) in 1.6Pt–W_18_O_49_ nanourchins, confirming the uniform Pt incorporation.

The SEM image of the 1.6Pt–W_18_O_49_ sample (Figure 1A) shows that it exhibited a relatively uniform morphology comprising a hierarchical urchin-like structure with an overall spherical shape. The nanourchins contained several branches made up of nanowires with an average length of approximately 249 nm and width of 21 ± 3 nm. TEM images for 1.6Pt–W_18_O_49_ (Figure 1B) confirm the presence of nanowires at the outermost surface of the Pt–W_18_O_49_ nanourchins. SEM and TEM images of all the Pt–W_18_O_49_ samples displayed similar morphological features, as illustrated in Figure S1A,B for 0.4Pt–W_18_O_49_ and 0.8Pt–W_18_O_49_, respectively. Interestingly, TEM images of the 1.6Pt–W_18_O_49_ sample revealed that the majority of the 1.6Pt–W_18_O_49_ nanowires were uniform in terms of size and shape and did not display Pt NPs at their surface. Only a small fraction of the nanourchins displayed Pt NPs on their surface as shown in Figure S2. This is also confirmed by STEM-HAADF, HRTEM, and STEM-EDS maps as shown in Figure 1C–E, respectively. A similar behavior was detected for 0.4Pt–W_18_O_49_ and 0.8Pt–W_18_O_49_ as shown in Figure S1C,D, respectively.

The HRTEM image of 1.6Pt–W_18_O_49_ nanowires (Figure 1D) revealed lattice fringe distances of 0.395 nm, which is slightly greater than that of the (010) plane of bare monoclinic W_18_O_49_, indicating that the Pt-modification leads to a change of the lattice parameter of W_18_O_49_. These results suggest that, in our experimental procedure, Pt may be doped into the W_18_O_49_ nanowires. STEM-HAADF images (Figure 1C) and the corresponding STEM-EDS maps of 1.6Pt–W_18_O_49_ (Figure 1E) show that W, O, and Pt were distributed homogeneously in the material, which further suggests that the Pt was incorporated via doping into W_18_O_49_. A similar behavior was also observed for 0.4Pt–W_18_O_49_ (Figure S1C) and 0.8Pt–W_18_O_49_ (Figure S1D). In agreement with previous reports, it is proposed that W_18_O_49_ nanourchins were formed via alcoholysis followed by hydrolysis of WCl_6_.^22^ In the presence of PtCl_4_^–^ this process also involves the reduction of PtCl_4_^–^ to Pt by 1-naphthol and ethanol, facilitating Pt incorporation into the W_18_O_49_ structure during the synthesis. 1-Naphthol was chosen herein due to its high solubility in ethanol and its ability to provide a slow reduction of PtCl_6_^2–^, potentially allowing for the uniform incorporation of Pt in the W_18_O_49_ structure.^23^

**Figure 2:**
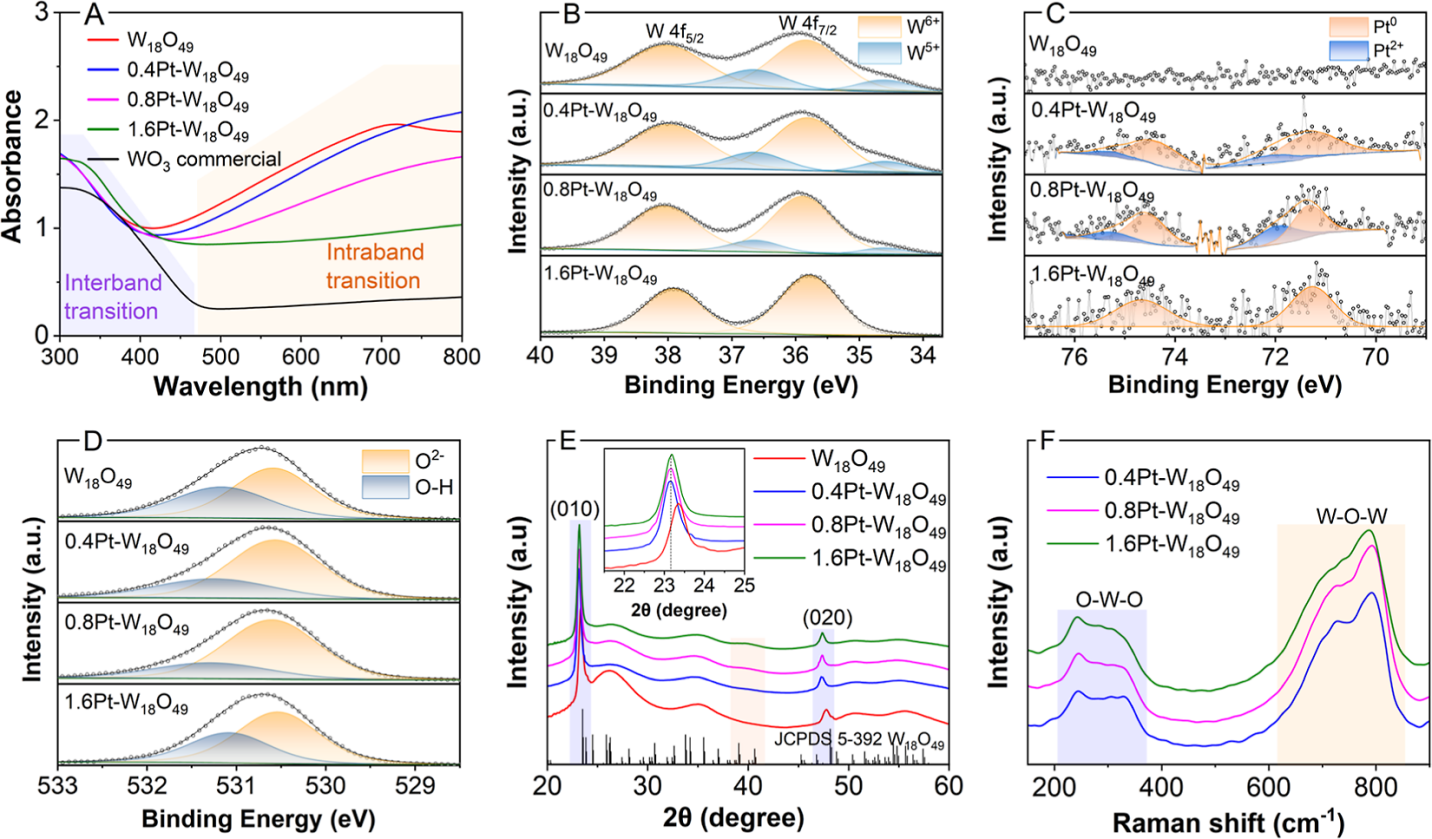
Optical, electronic, and structural characterization of Pt–W_18_O_49_ samples. (A) UV–vis absorption spectra of Pt–W_18_O_49_ samples (blue, magenta, and green traces) measured using an integrating sphere, highlighting interband (band gap excitation) and LSPR intraband transitions. The spectra for pure W_18_O_49_ (red trace) and commercial WO_3_ (black trace) are shown for comparison. High-resolution XPS spectra of the (B) W 4f, (C) Pt 4f, and (D) O 1s core levels for pure W_18_O_49_ (top panels) and Pt–W_18_O_49_ samples. It can be observed a shift in the W 4f binding energy to lower values and a decrease in the signals assigned to W^5+^ as well as an increase in the signals assigned to metallic Pt with the increase in Pt deposition in the W_18_O_49_ support. (E) XRD patterns and (F) Raman spectra for W_18_O_49_ (red trace in E) and Pt–W_18_O_49_ samples (blue, magenta, and green traces).

The optical properties of the Pt–W_18_O_49_ were investigated by UV–vis acquired using a diffuse reflectance integrating sphere. Undoped W_18_O_49_ exhibited a strong absorption band starting from 400 nm and extending to 800 nm (Figure 2A). This can be attributed to the localized surface plasmon resonance (LSPR) effect arising from the presence of W^5+^ sites and oxygen vacancies (V_o_).^24^ Additionally, the increased absorption below 400 nm is associated with the semiconductor band gap excitation. The absorption spectrum of the commercial WO_3_ (black line, Figure 2A) is shown for comparison. While it also possessed the band gap excitation from 480 to 300 nm, no LSPR absorption band was observed in the visible to near-IR (this sample is not expected to display LSPR excitation due to the absence of V_o_ and W^5+^ species). For the Pt–W_18_O_49_ samples, while a stronger LSPR band was observed for the 0.4Pt–W_18_O_49_ nanourchins, a decrease in the LSPR intensity was observed with increasing Pt content, following the order 0.4Pt–W_18_O_49_ > 0.8Pt–W_18_O_49_ > 1.6Pt–W_18_O_49_. This suggests that Pt incorporation/doping into the W_18_O_49_ may occur at V_o_ sites, leading to a decrease in the number of defects responsible for the LSPR in W_18_O_49_. These changes in optical properties are also illustrated by the color change of the Pt–W_18_O_49_ aqueous suspensions, from light blue to dark blue, with increasing Pt content, as shown in Figure S3.

The XPS survey spectra of 1.6Pt–W_18_O_49_ confirmed the presence of W and O (Figure S4). However, Pt was only detectable in the high-resolution Pt 4f spectrum, due to its low loading. For W_18_O_49_ (Figure 2B, top panel), the W 4f XPS spectrum was deconvoluted into two doublets. The first doublet corresponds to W^6+^ at binding energies of 35.9 and 38.1 eV, while the second doublet is correlated with W^5+^ at 34.6 and 36.7 eV.^25^ The analysis of the W 4f peak area revealed that the W_18_O_49_ was composed of 88% of W^6+^ and 12% W^5+^ (Table S2). This finding aligns with the previous reports on plasmonic W_18_O_49_, which attribute the LSPR in part to the presence of W^5+^ and V_o_.^24^ High-resolution W 4f spectra of 0.4Pt–W_18_O_49_ and 0.8Pt–W_18_O_49_ reveal two doublets, indicative of W^6+^ and W^5+^, consistent with pristine W_18_O_49_. Noticeably, the W^5+^ peak intensity decreases as Pt loading increases as depicted in Table S2. Finally, the W 4f high-resolution spectrum of 1.6Pt–W_18_O_49_ displays only a doublet corresponding to W^6+^ (35.7 and 37.9 eV), exhibiting a slight shift toward lower binding energy values. This observation agrees with the UV–vis and electron microscopy analysis and suggests a modification of the W_18_O_49_ electronic structure due to the Pt doping. Here, Pt donates electron density to W^6+^ species due to its metallic character and as W^6+^ exhibits higher electronegativity relative to Pt. This leads to a decrease in the binding energy.^26^ It was observed that increasing the Pt doping led to the oxidation of the W^5+^ sites to W^6+^. This agrees with the UV–vis results that showed decreased LSPR signals as the Pt content in the samples increased. When Pt is doped into W_18_O_49_, it may interact with or fill the V_o_ sites and leading to a reduction in the number of W^5+^ species and LSPR properties. Interestingly, our UV–vis results indicate that, while there was the complete disappearance of the LSPR peaks in 1.6Pt–W_18_O_49_, the samples 0.4Pt–W_18_O_49_ and 0.8Pt–W_18_O_49_ still displayed LSPR, which decreased in intensity with the increase in the Pt content. This suggests that doping at lower concentrations such as in 0.4Pt–W_18_O_49_ and 0.8Pt–W_18_O_49_ leads to only a partial suppression of the V_o_ and thus LSPR properties.

Figure 2C displays the Pt 4f spectra for the Pt–W_18_O_49_ samples. 0.4Pt–W_18_O_49_ and 0.8Pt–W_18_O_49_ exhibit two low-intensity doublets. These doublets correspond to Pt^0^ and Pt^2+^ oxidation states, with peaks at 71.3 eV (Pt^0^ 4f_7/2_), 72.2 eV (Pt^2+^ 4f_7/2_), 74.5 eV (Pt^0^ 4f_5/2_), and 75.3 eV (Pt^2+^ 4f_5/2_).^20^ The presence of Pt^2+^ in these samples is attributed to the low Pt concentration and doping, which favors the formation of Pt^2+^ and sites susceptible to PtO and Pt(OH)_2_ formation.^27^ In contrast, the 1.6Pt–W_18_O_49_ spectrum shows only two low-intensity peaks at 71.1 eV (Pt^0^ 4f_7/2_) and 74.6 eV (Pt^0^ 4f_5/2_), indicating the presence of exclusively metallic Pt.^28^ As expected, no Pt peaks were observed for pure W_18_O_49_ (Figure 2C, top panel).

The O 1s XPS spectra (Figure 2D) of both W_18_O_49_ and the Pt–W_18_O_49_ samples show only two peaks. One peak at 530.6 eV corresponds to the lattice oxygen (O^2–^) within the W–O–W bond, while a less intense peak at 531.2 eV can be attributed to hydroxyl groups at the surface (W–OH bond).^7,29^ No significant shifts or intensity ratio changes were observed as shown in Table S3.

Figure 2E shows the XRD patterns of both W_18_O_49_ and Pt–W_18_O_49_ samples. The XRD of undoped W_18_O_49_ (red trace) corresponded to the monoclinic crystal structure (JCPDS card 5–392). The intense and narrow peaks at 23.5 and 47.8° correspond to the (010) and (020) reflections of this monoclinic W_18_O_49_ phase, respectively. All the other secondary peaks were broad and had low intensity, suggesting preferential growth of the W_18_O_49_ crystals along the [010] direction, which agrees with the formation of nanowires.^30^ For the Pt–W_18_O_49_ samples, the XRD patterns were similar to those of the unmodified W_18_O_49_ samples (Figure 2E). Additionally, all the Pt–W_18_O_49_ samples exhibited a shift in peak positions toward smaller 2θ values. This shift indicates an expansion of the W_18_O_49_ lattice parameter because of the Pt doping, which is in agreement with our electron microscopy and spectroscopic data. Scherrer equation calculations (Table S4) showed an increase in the crystallite size with increasing Pt loading. Also, a broad peak emerged around 40° for the 1.6Pt–W_18_O_49_ sample. This peak can be attributed to the (111) reflection of nanocrystalline Pt, indicating the presence of Pt nanoparticles within the Pt–W_18_O_49_ samples. This suggests that in this sample, a mixture of Pt doping and Pt deposition at the surface occurred, which agrees with the electron microscopy results (Figure S2).

In the Raman spectra for the Pt–W_18_O_49_ samples (Figure 2F), the bands at low wavenumbers (between 208 and 370 cm^–1^, highlighted in blue) corresponds to the symmetric stretching of O–W–O bonds.^30^ A second, broad, and irregular band appears between 620 and 880 cm^–1^ (highlighted in orange). This band is assigned to the asymmetric stretching of W–O–W modes in the crystal structures of W_18_O_49_. No significant changes were observed in the spectra regardless of the Pt content.^30^

With a comprehensive understanding of the structural, optical, and electronic properties of the Pt–W_18_O_49_ nanourchins, we now turn our attention to the electrocatalytic performance of these materials in the HER as a model transformation. We aim to evaluate the influence of the Pt composition on the catalytic activity both under both dark and light excitation conditions. By comparing the HER performance across the different Pt–W_18_O_49_ compositions, we can gain insights into how the interplay between the Pt content and plasmonic effects impacts the overall efficiency of the catalyst.

**Figure 3:**
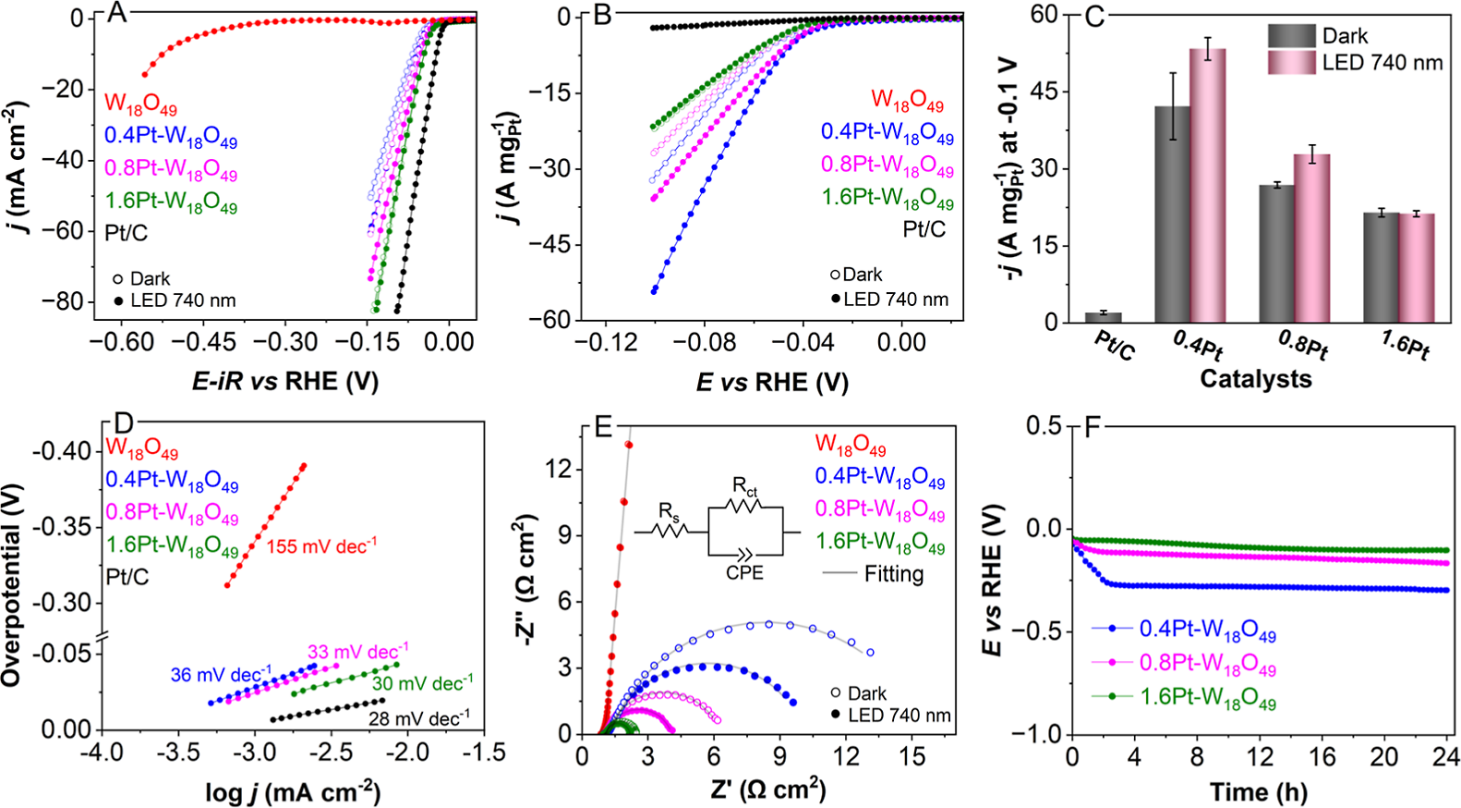
Electrocatalytic performance of W_18_O_49_ and Pt–W_18_O_49_ samples. (A) *iR* correct LSVs normalized by geometric area, (B) LSVs normalized by mass, (C) mass activity at −0.1 V_RHE_, (D) Tafel slopes, (E) EIS spectra obtained at −0.05 V_RHE_, and (F) chronopotentiometry (stability test) measured at −10 mA . All measurements were obtained in argon purged with 0.5 mol L^–1^ H_2_SO_4_ at 25 °C. The error bars in (C) were determined by performing experiments in triplicate.

Figure 3A presents the linear sweep voltammetry (LSV) curves (*iR*-corrected) measured at a scan rate of 5 mV s^–1^, measured under dark conditions and 740 nm LED illumination. Figure 3B shows the Pt mass-normalized LSVs and Figure 3C shows the corresponding mass activity at −0.1 V_RHE_. Among the tested catalysts, the undoped W_18_O_49_ nanourchins demonstrated the lowest activity, requiring an overpotential of 526 mV to achieve a current density of 10 mA cm^–2^. In contrast, the Pt-modified W_18_O_49_ catalysts showed significantly enhanced performance. 1.6Pt–W_18_O_49_ exhibited the best activity under the dark conditions, with an overpotential of only 45 mV at −10 mA cm^–2^, approaching the performance of commercial 20 wt.% Pt/C. The overpotentials for 0.4Pt–W_18_O_49_ and 0.8Pt–W_18_O_49_ were 66 mV and 58 mV, respectively. Under 740 nm illumination, a notable enhancement in the HER performance was observed for 0.4Pt–W_18_O_49_ and 0.8Pt–W_18_O_49_, which can be attributed to the LSPR excitation in these samples. Notably, a greater HER enhancement under the light excitation was observed for 0.4Pt–W_18_O_49_ than for 0.8Pt–W_18_O_49_, which agrees with the decrease in the LSPR band as the Pt content in the samples increased (Figure 2A). In this case, LSPR excitation can lead to the formation of hot electrons that migrate to active Pt sites, improving the HER kinetics. Importantly, LSPR excitation can also lead to localized photothermal heating, which can enhanced HER kinetics and these effects are difficult to precisely disentangle under our conditions.^31−33^ Moreover, 1.6Pt–W_18_O_49_ did not exhibit light-enhanced activity, correlating with UV–vis and XPS analyses (Figure 2A,B), which revealed the disappearance of the LSPR band and the absence of W^5+^ species necessary for the LSPR effect as the Pt content was the highest. The comparison of uncorrected and *iR*-corrected LSVs of 1.6Pt–W_18_O_49_ (Figure S5) highlights that correcting for *iR* losses leads to slightly improved HER performance. The Pt-mass-normalized LSVs (Figure 3B) emphasizes the superior mass activity of all the Pt–W_18_O_49_ catalysts over Pt/C. As shown in Figure 3C, the mass activity at −0.1 V of 0.4Pt–W_18_O_49_ surpassed that of Pt/C by factors of 15 and 30 in the dark and under light illumination, respectively. Similarly, 0.8Pt–W_18_O_49_ displayed 13- and 17-fold enhancements over Pt/C under the dark and illuminated conditions. 1.6Pt–W_18_O_49_ exhibited a 10-fold activity enhancement compared to Pt/C under dark conditions. However, no such enhancement was observed under the LED illumination, aligning with UV–vis and XPS data suggesting suppressed LSPR effects in this material. Table S5 summarizes the HER performance of the state-of-the-art noble and non-noble metal-based electrocatalysts in acidic electrolytes, revealing that 1.6Pt–W_18_O_49_ is among the most active materials for HER. These results underscore the exceptional electrocatalytic performance of the Pt–W_18_O_49_ catalysts, characterized by low onset potentials, low overpotentials, and high current densities.

To accurately assess the intrinsic catalytic activity and eliminate surface-area-specific variations, the current density from the linear sweep voltammograms (LSVs, Figure 3A) was normalized by the electrochemical surface area (ECSA). The ECSA was determined using double-layer capacitance (*C*_dl_) values, extracted from cyclic voltammetry measurements (Figure S6A–D). Specifically, *C*_dl_ was calculated from the slope of the linear relationship between the average current density (Δ*j*) and the scan rate (Figure S6E). The resulting ECSA (Figure S6F) and ECSA-normalized LSVs (Figure S7) reveal that all the Pt–W_18_O_49_ catalysts outperform the benchmark Pt/C, underscoring their superior intrinsic activity.

Tafel slopes, which provide insights into the reaction kinetics and the HER mechanism, were derived from the LSV curves in Figure 3A and are summarized in Figure 3D. The 1.6Pt–W_18_O_49_ catalyst exhibits a low Tafel slope of 30 mV dec^–1^, indicating that desorption of H_2_ is the rate-limiting step, with the HER proceeding via the Volmer–Tafel pathway (eqs 1 and 2). For comparison, the commercial Pt/C catalyst shows a Tafel slope of 28 mV dec^–1^, which is consistent with the reported values. Similarly, the 0.8Pt–W_18_O_49_ and 0.4Pt–W_18_O_49_ catalysts exhibit Tafel slopes of 33 and 36 mV dec^–1^, respectively, confirming the predominance of the Volmer–Tafel mechanism in these systems as well. In stark contrast, the pristine W_18_O_49_ material shows a significantly higher Tafel slope of 155 mV dec^–1^, reflecting sluggish kinetics and a less efficient HER process. These data suggests that the Pt sites are primarily responsible for the catalytic activity. While W_18_O_49_ may play a supporting role, it likely does not directly participate in the hydrogen evolution process.
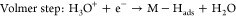
1
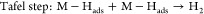
2

To gain deeper insight into the charge transfer dynamics at the W_18_O_49_ interface, electrochemical impedance spectroscopy (EIS) was conducted under both dark and illuminated conditions (Figure 3E). The Nyquist plots exhibit a single semicircle, which is consistent with a single charge-transfer process, and aligns well with the HER mechanism inferred from the Tafel slopes. The impedance data were analyzed using a Randles equivalent circuit model (inset, Figure 3E), comprising a series resistance (*R*_s_) of the electrolyte, *C*_dl_, and a charge transfer resistance (*R*_ct_) reflecting the electron transfer rate between the electrode and reactive species. The extracted parameters are summarized in Table S6. Among the tested catalysts, 1.6Pt–W_18_O_49_ displayed the lowest *R*_ct_ values under both dark (10.5 Ω cm^2^) and illuminated (9.2 Ω cm^2^) conditions. These nearly identical *R*_ct_ values suggest that the LSPR effect has a minimal impact on this catalyst. In contrast, 0.4Pt–W_18_O_49_ and 0.8Pt–W_18_O_49_ showed marked decreases in *R*_ct_ upon illumination, underscoring the role of LSPR in enhancing the charge transfer. The lower *R*_ct_ value seen for 1.6Pt–W_18_O_49_ is in line with its superior activity per geometric electrode area, as reflected in Figure 3A. By contrast, the 0.4Pt–W_18_O_49_ and 0.8Pt–W_18_O_49_ catalysts deliver higher mass-normalized activity (Figure 3B), but *R*_ct_ primarily gauges the resistance to charge transfer at the electrode’s surface, rather than correlating with the mass-based activity. Regarding pristine W_18_O_49_, its Nyquist plot has now been included in Figure 3E to illustrate how charge transfer resistance in the undoped oxide compares to the Pt-modified samples.

The electrochemical stability of the Pt–W_18_O_49_ catalysts during sustained HER was evaluated using chronopotentiometry at a current density of −10 mA cm^–2^ in 0.5 mol L^–1^ H_2_SO_4_ (Figure 3F). 0.4Pt–W_18_O_49_ exhibited an initial decline in the current density over the first 4 h, indicative of deactivation, followed by stable performance. This deactivation likely stems from structural instability and Pt aggregation, which are attributable to the low Pt loading in this catalyst. A similar but less pronounced trend was observed with 0.8Pt–W_18_O_49_. Notably, 1.6Pt–W_18_O_49_ demonstrated the best stability, maintaining consistent performance throughout the 24 h test. Poststability SEM analysis confirmed that the morphology of 1.6Pt–W_18_O_49_ remained largely intact (Figure S8), highlighting its excellent structural integrity.

Our characterization data indicate that, while 1.6Pt–W_18_O_49_ exhibits both doped Pt and surface Pt clusters, 0.4Pt–W_18_O_49_ and 0.8Pt–W_18_O_49_ contain Pt primarily in a doped or highly dispersed state (Figures S1 and S2). The TEM images (Figures 1, S1 and S2) show the number and size of visible Pt clusters at the surface diminish as the Pt content decreases, consistent with doping rather than substantial surface deposition. These results are supported by the XRD patterns (Figure 2E), where increasing Pt peaks appear in higher-loading samples, and by new XPS data (Figure 2C), revealing partially oxidized Pt^2+^ species in 0.4Pt–W_18_O_49_ and 0.8Pt–W_18_O_49_. These findings align with earlier work showing that low Pt concentrations favor dispersed Pt and partial oxidation.^34^

Although both doping and surface Pt deposition occur, 1.6Pt–W_18_O_49_, which displays superior stability (Figure 3F), evidently contains a higher fraction of metallic Pt clusters. This suggests a trade-off between activity and stability. Doped Pt in 0.4Pt–W_18_O_49_ and 0.8Pt–W_18_O_49_ leads to higher mass activity through a greater number of accessible active sites per unit mass of Pt and partial LSPR enhancement. However, stability may suffer at lower Pt loadings if fewer metallic clusters are available to endure extended electrochemical cycling. The 0.4Pt–W_18_O_49_ and 0.8Pt–W_18_O_49_ samples contain fewer Pt sites that are mostly doped onto the W_18_O_49_ structure or highly dispersed at the surface according to our characterization data. This can make the Pt more prone to migration and aggregation under electrochemical conditions by favoring localized restructuring or dissolution–redeposition processes.^35,36^ By contrast, the higher Pt loading in 1.6Pt–W_18_O_49_ provides a greater density of larger Pt NPs at the surface (12 nm), helping mitigate structural reorganization during prolonged operation. Consequently, the 1.6Pt–W_18_O_49_ catalyst experiences less deactivation, as manifested by its lower drop in current density over the same time frame relative to other samples. Thus, our findings highlight the complementary roles of doped Pt and surface Pt in driving the HER, where doping contributes to high intrinsic activity and synergy with plasmonic effects, while larger metallic clusters can better maintain long-term performance.

To explore the effect of lower Pt content, we synthesized two additional W_18_O_49_ samples containing 0.1% and 0.2% Pt (0.1Pt–W_18_O_49_ and 0.2Pt–W_18_O_49_ respectively; Figure S9). Although 0.2Pt–W_18_O_49_ displayed higher mass activity compared to 0.1Pt–W_18_O_49_ (and all other materials tested), its HER performance under illumination did not approach the substantial light-driven enhancement observed for 0.4Pt–W_18_O_49_. Reducing the Pt loading below 0.4 wt % appears to limit the availability of active sites for electron transfer, and the resulting LSPR enhancement from W_18_O_49_ alone cannot fully compensate. Indeed, 0.1Pt–W_18_O_49_ exhibited markedly lower mass activity, confirming that insufficient Pt compromises catalytic performance. Although the 0.2Pt–W_18_O_49_ sample initially performed well, we anticipate poor stability—akin to the partial deactivation observed in 0.4Pt–W_18_O_49_ (Figure 3F). Thus, while decreasing Pt content can strengthen LSPR intensity, maintaining an adequate density of Pt sites is critical for robust and stable HER activity. Our data therefore suggest that lowering the Pt content much below 0.4 wt % does not reliably translate into improved HER performance, despite the associated increase in LSPR intensity.

**Figure 4:**
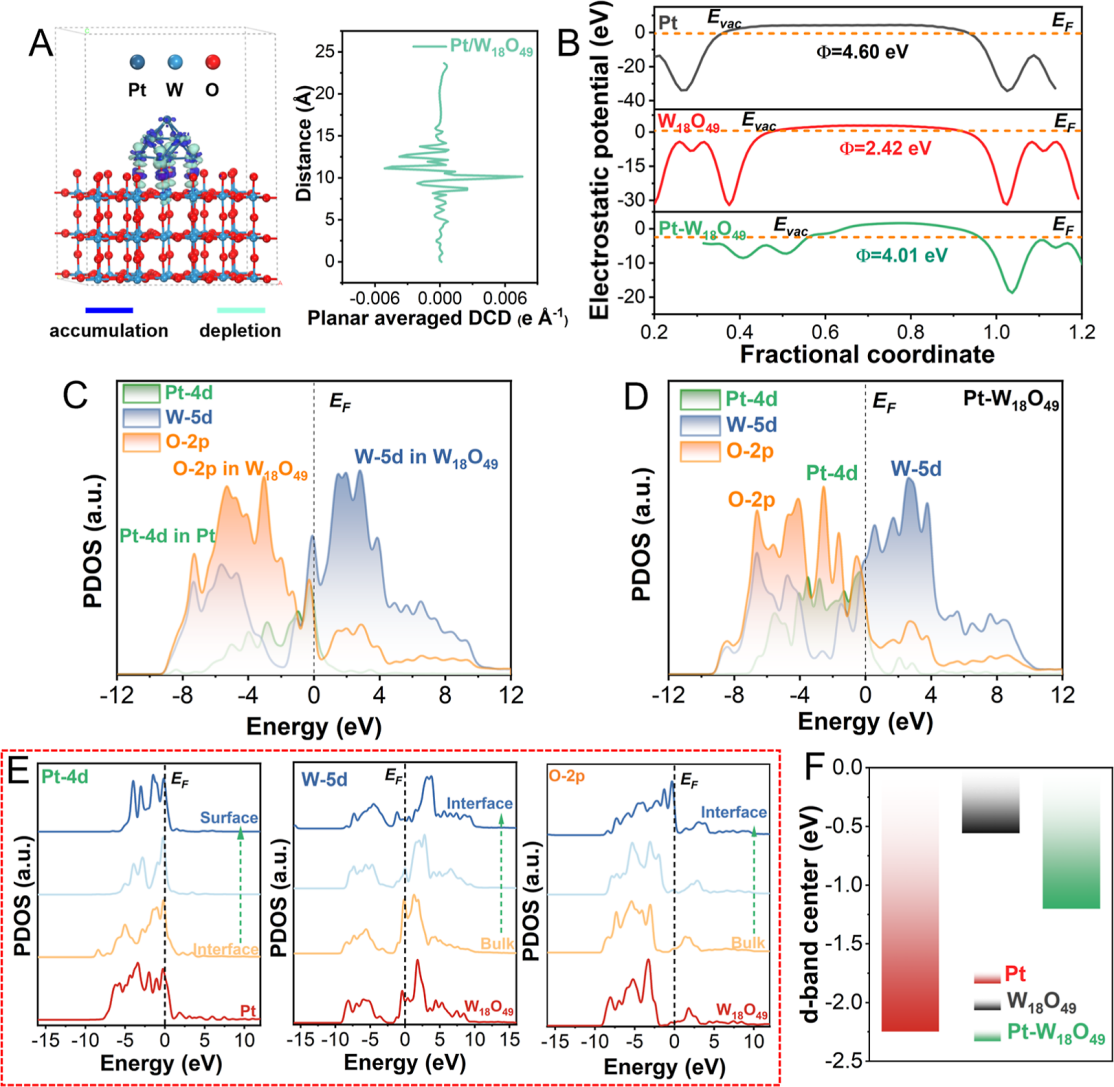
Electronic structure and charge distribution analysis of Pt–W_18_O_49_. (A, left panel) charge density differences in the constructed Pt–W_18_O_49_ model (side view). The blue and cyan contours represent the regions of electron accumulation and depletion, respectively, where the isosurfaces are set to 0.05 e Å^–3^. (A, right panel). The plane averaged differential charge density (DCD) across the interface. (B) Electrostatic potentials for Pt(111), W_18_O_49_, and Pt–W_18_O_49_ models. Projected density of states (PDOS) curves for (C) Pt(111) and W_18_O_49_ and (D) Pt–W_18_O_49_. (E) Site-dependent PDOS of Pt 4d, W 5d, and O 2p. (F) d-band centers of Pt(111), W_18_O_49_, and Pt–W_18_O_49_ models.

Density functional theory (DFT) calculations were conducted to elucidate the mechanism behind the enhanced HER activity of Pt–W_18_O_49_. Models representing Pt–W_18_O_49_, Pt(111), and W_18_O_49_ were constructed (Figure S10). The charge density difference (CDD) analysis (Figure 4A, left panel) revealed significant charge redistribution at the Pt–W_18_O_49_ interface. This redistribution featured areas of electron accumulation (blue) and depletion (cyan), highlighting a robust interaction between the Pt clusters and W_18_O_49_. Such a configuration likely facilitates the migration of hot charges generated by the LSPR effect to Pt, enhancing electron transfer efficiency during the HER.^37^ The planar average differential charge density (DCD) (Figure 4A, right panel) further confirmed this redistribution, suggesting the formation of a built-in electric field at the interface. This was supported by work function (*W*_F_) calculations, which showed a smaller *W*_F_ for W_18_O_49_ compared to Pt(111). This reduction reflects a higher Fermi energy level for W_18_O_49_, implying that charge redistribution equilibrates the work functions of Pt and W_18_O_49_, optimizing electron transfer across the interface (Figure 4B).

Loading Pt clusters also induced significant changes in the electronic structure of Pt–W_18_O_49_. Compared to the isolated Pt and W_18_O_49_ components, the Pt 4d orbitals exhibited stronger interactions, including d–d coupling with W 5d orbitals and d–p coupling with O 2p orbitals (Figure 4C,D). These couplings enhance electron transfer efficiency and stabilize the metal valence states. Interestingly, the Pt 4d orbitals were found deep below the Fermi level (*E*_F_), serving as an electron reservoir, while the W 5d orbitals, partially above *E*_F_, demonstrated a prominent peak near it, enabling efficient electron depletion. These features synergize to promote the HER activity. Projected partial density of states (PDOS) analyses (Figure 4E) further clarified the roles of different orbitals. The Pt 4d orbitals showed enhanced electroactivity from the interface to the surface, while the W 5d orbitals shifted away from the Fermi level with broadened bands from the bulk to the interface. This complementary PDOS trend ensures high HER activity at the interface.^38^ Similarly, the O 2p orbitals exhibited an upward shift, improving electrical conductivity at the surface oxygen sites. Finally, the d-band center (*d*_c_) analysis revealed that the moderate *d*_c_ position of W_18_O_49_ stems from electronic modulation by Pt clusters. This adjustment fine-tunes the adsorption characteristics, further boosting the HER performance (Figure 4F). Together, these findings underscore the intricate synergy between Pt and W_18_O_49_ in enhancing the HER efficiency through electronic structure optimization and effective charge redistribution.

**Figure 5:**
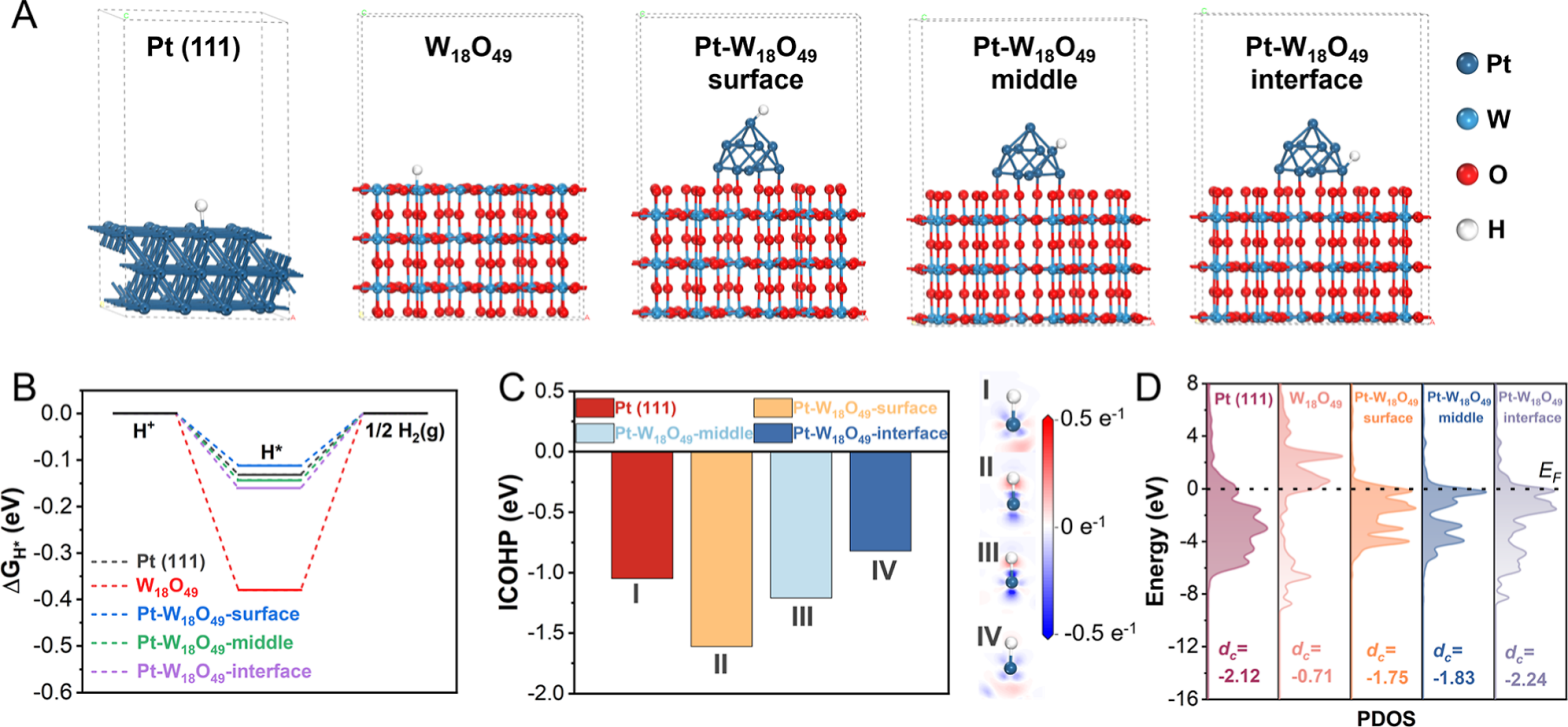
Hydrogen adsorption and electronic properties of Pt–W_18_O_49_ for the HER. (A) Possible hydrogen-adsorption sites over Pt(111), W_18_O_49_, and Pt–W_18_O_49_ surface/middle/interface regions. (B) Gibbs free energy (Δ*G*_H*_) profiles for the HER. (C) The integrated crystal orbital Hamilton population (ICOHP) of various M–H configurations and the 2D electron density differences after adsorption (right panel). Bule and red represent the depletion and accumulation of electrons, respectively, in units of e Å^–3^. (D) d-PDOS on various catalytic sites.

From an energetic perspective, Gibbs free energies (Δ*G*_H*_) were calculated for various possible active sites, including Pt in Pt(111), W in W_18_O_49_, and Pt in Pt–W_18_O_49_ at the surface, middle, and interface regions (Figure 5A). The results (Figure 5B) showed that all calculated Δ*G*_H*_ values were negative, confirming favorable conditions for hydrogen adsorption (H*) and desorption (H_2_). Among the sites studied, the Pt–W_18_O_49_ surface exhibited the lowest Δ*G*_H*_ value, indicating that the combination of Pt clusters and W_18_O_49_ creates abundant active sites optimized for the HER kinetics. This synergy facilitates both hydrogen adsorption and its efficient release as molecular hydrogen.

To further probe the bonding characteristics at the Pt–H sites, crystal orbital Hamilton population (COHP) analysis was performed alongside a two-dimensional (2D) CDD analysis of the metal–hydrogen (M–H) configuration. The integrated COHP (ICOHP) values, derived by summing the bonding contributions below the Fermi level, revealed stronger bonding at the Pt–W_18_O_49_ surface than the other configurations did (Figure 5C). These findings were corroborated by the 2D-CDD maps, which displayed pronounced charge transfer in the Pt–H bonds at the Pt–W_18_O_49_ surface, highlighting its superior bonding capability. Interestingly, *d*_c_ analyses of the Pt sites at the Pt–W_18_O_49_ middle and interface regions highlighted their favorable electronic configurations for releasing hydrogen as H_2_ (Figure 5D). This balance between the strong adsorption at the surface and efficient desorption from deeper layers indicates that the Pt–W_18_O_49_ system achieves an optimal electronic structure for the HER catalysis. Overall, the interplay of Pt and W_18_O_49_ results in a finely tuned electronic environment, providing moderate adsorption and desorption characteristics that significantly enhance HER activity.

## Conclusions

In this study, we successfully developed Pt–W_18_O_49_ as an active electrocatalyst for the hydrogen evolution reaction (HER). Using a one-step solvothermal synthesis, we achieved ultralow Pt loadings while maintaining exceptional catalytic performance. Among the tested catalysts, Pt–W_18_O_49_ with 0.4 wt % Pt demonstrated outstanding mass activity, surpassing commercial Pt/C by 15-fold under dark conditions and 30-fold under visible light illumination. The 1.6 wt % Pt–W_18_O_49_ catalyst exhibited highest performance in terms of stability, further underscoring the versatility of this system. These results establish Pt–W_18_O_49_ as a promising candidate for HER catalysis, with additional activation enabled by visible light through plasmonic excitation.

The remarkable catalytic performance stems from the combination between the W_18_O_49_ support and Pt sites. Localized surface plasmon resonance (LSPR) in W_18_O_49_ enhances charge transfer, promotes hot electron generation, and optimizes hydrogen adsorption–desorption dynamics. Density functional theory (DFT) calculations revealed that the Pt–W_18_O_49_ interface fosters charge redistribution, strong orbital interactions, and a favorable electronic structure. This combination fine-tunes adsorption and desorption energies and improves HER kinetics. Furthermore, Pt incorporation modulates the d-band center of Pt–W_18_O_49_, enhancing electron transfer and catalytic efficiency. This work highlights Pt–W_18_O_49_ as an innovative platform to minimize Pt usage while maximizing catalytic performance. Beyond its practical application, it offers valuable insights into designing plasmon-enhanced electrocatalysts for sustainable hydrogen production. We hope these findings inspire new research directions in light-responsive catalysis, contributing to the global pursuit of clean energy solutions.

## Methods

### Materials and Instrumentation

Hexachloroplatinic acid hexahydrate (H_2_PtCl_6_·6H_2_O), tungsten chloride (WCl_6_), α-naphthol and 20 wt % Pt/C were purchased from Merck. Absolut ethanol was purchased from the Thermo Scientific. All the chemicals were used without further purifications. Deionized water (18.2 MΩ·cm Milli-Q system) was used for synthesis and to support electrolyte preparation.

### Material Characterization

A UV–VIS spectrophotometer (Shimadzu UV–2600) equipped with an integrating sphere (ISR 2600) was used to record the UV–vis absorption spectra by diffuse reflectance spectroscopy (DRS) from the powder samples. Microwave plasma atomic emission spectroscopy (MP-AES) (Agilent Technologies MP–AES 4100 spectrometer) was used to quantify the platinum content. All solutions for MP-AES were prepared using a 0.5 M HCl solution. Platinum standards of 1, 2, 5, 7, and 10 ppm for the equipment calibration were prepared by the dilution of a 1000 ppm Pt standard solution for AAS (Sigma-Aldrich). The catalysts analysis solutions were prepared by dissolving 5 mg of the catalyst in 4 mL of aqua regia. After evaporation part of the liquid, the catalyst was dispersed in 10 mL of HCl solution and the undissolved W_18_O_49_ was separated by centrifugation (7000 rpm, 10 min).

Scanning electron microscopy (SEM) images were obtained using a Hitachi S–4800 field emission microscope, operating at an accelerating voltage of 30 kV. SEM samples were prepared by dispersing the nanoparticle suspension in ethanol–water with an ultrasonic bath and drop casting onto a Si wafter, followed by drying under ambient conditions before imaging. Transmission electron microscopy (TEM) images were obtained on a Jeol JEM-1400 TEM, operating at an accelerating voltage of 120 kV. High resolution bright field and dark field images were taken with a HD-2700 200 kV Cs-corrected STEM. Furthermore, the high-angle annular dark field (HAADF) and composition examinations were conducted on Hitachi SU9000 operating at 30 kV equipped with energy dispersive spectroscopy (EDS) detector (Oxford Ultim Extreme). (S)TEM samples were prepared by dispersing the nanoparticle suspension in ethanol with an ultrasonic bath and drop casting onto carbon-coated copper grids.

X–ray photoelectron spectroscopy (XPS) was performed using a PREVAC spectrometer with a monochromatized Al Kα anode (1486.7 eV) under ultrahigh vacuum (10^–10^ mbar). Survey spectra were measured with 200 eV pass energy (slit s 2.5 × 25) and high–resolution spectra were measured with 100 eV pass energy (slit c 0.8 × 25).Such configuration is enabling acquisition of a fwhm 0.61 eV for the Ag 3d at PE 100 eV. Casa XPS software was used for data interpretation. To investigate the crystal structure, X-ray diffraction was performed using a PANalytical X’Pert PRO diffractometer equipped with a Cu K_α1_ X-ray source, working at 45 kV and beam current fixed at 40 mA. X-ray diffractograms were obtained with a scanning rate of 0.2° min^–1^ and a step size of 0.02°. The sample was measured in the 2θ range from 20 to 60^ο^. Raman spectra were recorded using a Bruker FT-Raman RAM II spectrometer equipped with a 633 nm laser and working with an exposure time of 1 s.

### Synthesis of Pt-Supported W_18_O_49_ Nanourchins (Pt–W_18_O_49_)

The Pt–W_18_O_49_ samples were synthesized through a solvothermal method using WCl_6_ and H_2_PtCl_6_·6H_2_O as precursors. Typically, 100 mg of WCl_6_ was dissolved in 30 mL of absolute ethanol for 30 min under stirring resulting in a transparent yellow solution. After that, 0.227, 0.454, or 908 mL of 7 mmol L^–1^ H_2_PtCl_6_ and 1 mL of 0.5 mol L^–1^ α-naphthol were added in the WCl_6_ solution. After 5 min, the solution was transferred into a 45–mL Teflon–lined autoclave, which was sealed and heated at 180 °C for 24 h using a heating rate of 3 °C/min. After the autoclave cooled down to room temperature naturally, the product was collected by centrifugation and washed with absolute ethanol for 4 times. Finally, it was dried at 50 °C in an oven overnight.

### Electrochemical Characterization

Electrochemical measurements were performed using an Autolab PGSTAT128N potentiostat in a standard three-electrode cell configuration. A 4.0 mm diameter L-shaped glassy carbon electrode (geometric area: 0.1257 cm^2^) served as the working electrode, a graphite rod as the counter electrode, and a reversible hydrogen electrode (RHE) as the reference electrode. Catalyst inks were prepared by dispersing 3.0 mg of catalyst in an ethanol/water/Nafion 5% (7:2.9:0.1 v/v/v) solution and sonicating for 60 min to obtain a homogeneous mixture. Subsequently, approximately 8.4 μL of the ink was drop-casted onto the glassy carbon electrode and dried at 50 °C, resulting in a catalyst loading of 200 μg cm^–2^. Linear sweep voltammograms were acquired in the dark and under 740 nm light-emitting diode (LED) illumination (72 mW cm^–2^) from 0 to −0.2 V at 5 5 mV s^–1^. Stability tests were performed by chronopotentiometry at −10 mA cm^–2^ for 24 h. For the stability tests the catalyst loading used was 400 μg cm^–2^. The electrochemical impedance spectroscopy was measured in potential mode at −0.05 V from 100 kHz to 10 Hz with an amplitude of 10 mV. All the electrochemical measurements were performed in 0.5 mol L^–1^ H_2_SO_4_ solution as the electrolyte.

### Computational Methods

DFT calculations were performed with the first-principles simulation Cambridge sequential total energy package (CASTEP) module in Materials Studio software.^39^ The exchange–correlation potential was described by the generalized gradient approximation (GGA) with the Perdewe–Burkee–Ernzerhof (PBE) functional.^40^ The interactions between valence electrons and ionic cores were described by the OTFG ultrasoft pseudopotential method. A plane-wave basis set with a cutoff energy of 380 eV was assigned to the potential method. The empirical dispersion correction in Grimme’s scheme was employed to consider the van der Waals (vdW) interaction. The linear Broyden–Fletcher–Goldfarb–Shanno (LBFGS) algorithm with a medium quality setting of *k*-points was used for all the energy minimizations in this work. The geometry optimization convergence tolerances for the energy change, maximum force and maximum displacement were 5 × 10^–5^ eV/atom, 0.001 eV/Å, and 0.005 Å, respectively.

We constructed the Pt–W_18_O_49_ models by combining a Pt cluster with the W_18_O_49_ (010) surface, based on our XRD data indicating monoclinic W_18_O_49_ and HRTEM observations revealing preferential crystal growth along the [010] direction. In preparing the model, we used a three-layer unit cell, with lattice parameters of 18.27 and 14.19 Å, to capture the characteristic structural features. A 20 Å vacuum gap was introduced along the *z*-axis to prevent interslab interactions during geometry optimization. We then placed a 10-atom Pt cluster on the W18O_49_ (010) surface, which accords with our experimental findings suggesting both doped and surface-bound Pt.^41^ During energy minimization, the bottom layer was fixed to preserve the bulk-like configuration of W_18_O_49_, while the Pt cluster and the top two layers of W_18_O_49_ were allowed to fully relax. This approach accounts for the local electronic and geometric rearrangements at the Pt–W_18_O_49_ interface, reflecting the interfacial phenomena that likely occur in the actual catalyst. Consequently, the final model provides a realistic representation of how Pt might anchor onto W_18_O_49_, enabling us to connect our theoretical insights with the experimentally observed catalytic behavior.

The Gibbs free energies for hydrogen adsorption (Δ*G*_H*_) were calculated from the following equation
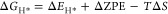
3where Δ*E*_H*_, ΔZPE, *T* and Δ*S* represent the binding energy, zero-point energy change, temperature, and entropy change of the H* adsorption system, respectively. It is generally considered that the vibrational entropy of H* in the adsorbed state is negligible. Thus, Δ*S* can be obtained from the following equation
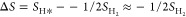
4where *S*_H_2__ is the entropy of H_2_ in the gas phase under the standard conditions. In addition, ΔZPE can be calculated by the following equation
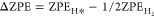
5

Hence, the free energy of the adsorbed state can be calculated using the following simplified equation: Δ*G*_H*_ = Δ*E*_H*_ + 0.24 eV.

## Supporting Information Available

Additional characterization (MP-AES, photographs, XPS, LSVs); catalytic data (LSVs, EIS data, and comparison table); and computational modeling (PDF)

## References

[ref1] Das S.; Peter S. C. Green Hydrogen from Wastewater–A Dual Crisis Resolution. Energy Fuels 2024, 38 (18), 17297–17308. 10.1021/acs.energyfuels.4c03122.

[ref2] Boettcher S. W. Introduction to Green Hydrogen. Chem. Rev. 2024, 124 (23), 13095–13098. 10.1021/acs.chemrev.4c00787. 39659176

[ref3] Terlouw T.; Rosa L.; Bauer C.; McKenna R. Future Hydrogen Economies Imply Environmental Trade-Offs and a Supply-Demand Mismatch. Nat. Commun. 2024, 15 (1), 7043 10.1038/s41467-024-51251-7. 39147777 PMC11327350

[ref4] Cheng Q.; Hu C.; Wang G.; Zou Z.; Yang H.; Dai L. Carbon-Defect-Driven Electroless Deposition of Pt Atomic Clusters for Highly Efficient Hydrogen Evolution. J. Am. Chem. Soc. 2020, 142 (12), 5594–5601. 10.1021/jacs.9b11524. 32088958

[ref5] Zhang T.; Wu M. Y.; Yan D. Y.; Mao J.; Liu H.; Hu W.-B.; Du X. W.; Ling T.; Qiao S. Z. Engineering Oxygen Vacancy on NiO Nanorod Arrays for Alkaline Hydrogen Evolution. Nano Energy 2018, 43, 103–109. 10.1016/J.NANOEN.2017.11.015.

[ref6] Cheng N.; Stambula S.; Wang D.; Banis M. N.; Liu J.; Riese A.; Xiao B.; Li R.; Sham T. K.; Liu L. M.; Botton G. A.; Sun X. Platinum Single-Atom and Cluster Catalysis of the Hydrogen Evolution Reaction. Nat. Commun. 2016, 7, 13638 10.1038/ncomms13638. 27901129 PMC5141386

[ref7] Cai J.; Javed R.; Ye D.; Zhao H.; Zhang J. Recent Progress in Noble Metal Nanocluster and Single Atom Electrocatalysts for the Hydrogen Evolution Reaction. J. Mater. Chem. A 2020, 8 (43), 22467–22487. 10.1039/D0TA06942F.

[ref8] Krishnan S.; Koning V.; Theodorus de Groot M.; de Groot A.; Mendoza P. G.; Junginger M.; Kramer G. J. Present and Future Cost of Alkaline and PEM Electrolyser Stacks. Int. J. Hydrogen Energy 2023, 48 (83), 32313–32330. 10.1016/j.ijhydene.2023.05.031.

[ref9] Shi G.; Tano T.; Tryk D. A.; Iiyama A.; Uchida M.; Terao K.; Osada H.; Yamaguchi M.; Tamoto K.; Kakinuma K. Nanostructured Pt-NiFe Oxide Catalyst for Hydrogen Evolution Reaction in Alkaline Electrolyte Membrane Water Electrolyzers. ACS Catal. 2024, 14 (12), 9460–9468. 10.1021/acscatal.4c01685.

[ref10] Xu Y.; Yu S.; Tong F.; Wang Z.; Wang P.; Liu Y.; Cheng H.; Fan Y.; Wei W.; Dai Y.; Zheng Z.; Huang B. Dual-Plasmon-Enhanced Nitrophenol Hydrogenation over W18O49-Au Heterostructures Studied at the Single-Particle Level. Catal. Sci. Technol. 2023, 13 (5), 1301–1310. 10.1039/D2CY02071H.

[ref11] Tang N.; Liu D.; Chen S.; Wang Z.; Ma Y.; Li Q.; Li Y.; Xu G.; Wu C.; Kang L.; Luo W.; Qiao B.; Zhu H.; Cong Y. Pt Atom-Substituted MoC Single-Atom Catalyst for Enhancing H2 Production. ACS Catal. 2024, 14 (19), 14297–14307. 10.1021/acscatal.4c01821.

[ref12] Zhang Y.-C.; Zhao M.; Wu J.; Wang Y.; Zheng L.; Gu F.; Zou J.-J.; Gao J.; Zhu X.-D. Construction of Pt Single-Atom and Cluster/FeOOH Synergistic Active Sites for Efficient Electrocatalytic Hydrogen Evolution Reaction. ACS Catal. 2024, 14 (10), 7867–7876. 10.1021/acscatal.4c01133.

[ref13] Wu R.; Zhang J.; Shi Y.; Liu D.; Zhang B. Metallic WO2-Carbon Mesoporous Nanowires as Highly Efficient Electrocatalysts for Hydrogen Evolution Reaction. J. Am. Chem. Soc. 2015, 137 (22), 6983–6986. 10.1021/jacs.5b01330. 25992910

[ref14] Jaramillo T. F.; Jørgensen K. P.; Bonde J.; Nielsen J. H.; Horch S.; Chorkendorff I. Identification of Active Edge Sites for Electrochemical H2 Evolution from MoS2 Nanocatalysts. Science 2007, 317 (5834), 100–102. 10.1126/science.1141483. 17615351

[ref15] Jia H.; Meng L.; Lu Y.; Liang T.; Yuan Y.; Hu Y.; Dong Z.; Zhou Y.; Guan P.; Zhou L.; Liu C.; Li M.; Wan T.; Ni B.-J.; Han Z.; Chu D. Boosting the Efficiency of Electrocatalytic Water Splitting via in Situ Grown Transition Metal Sulfides: A Review. J. Mater. Chem. A 2024, 12 (42), 28595–28617. 10.1039/D4TA06197G.

[ref16] Zhang H.; Wang Y.; Zuo S.; Zhou W.; Zhang J.; Lou X. W. D. Isolated Cobalt Centers on W18O49 Nanowires Perform as a Reaction Switch for Efficient CO2 Photoreduction. J. Am. Chem. Soc. 2021, 143 (5), 2173–2177. 10.1021/jacs.0c08409. 33508937

[ref17] Jones D. R.; Gomez V.; Bear J. C.; Rome B.; Mazzali F.; McGettrick J. D.; Lewis A. R.; Margadonna S.; Al-Masry W. A.; Dunnill C. W. Active Removal of Waste Dye Pollutants Using Ta3N5/W18O49 Nanocomposite Fibres. Sci. Rep. 2017, 7 (1), 4090 10.1038/s41598-017-04240-4. 28642612 PMC5481444

[ref18] Yang R. H.; Chueh L. Y.; Liao S. L.; Pan Y. T. Synthesis of Plasmonic W18O49 Interconnected Nanowire Frameworks with Strong Photo-Enhanced Hydrogen Evolution Reaction Activity. Mater. Today Sustain. 2023, 24, 100485 10.1016/j.mtsust.2023.100485.

[ref19] Yan T.; Chen S.; Sun W.; Liu Y.; Pan L.; Shi C.; Zhang X.; Huang Z. F.; Zou J. J. IrO2 Nanoparticle-Decorated Ir-Doped W18O49 Nanowires with High Mass Specific OER Activity for Proton Exchange Membrane Electrolysis. ACS Appl. Mater. Interfaces 2023, 15 (5), 6912–6922. 10.1021/acsami.2c20529. 36718123

[ref20] Bezerra L. S.; Brasseur P.; Sullivan-Allsop S.; Cai R.; da Silva K. N.; Wang S.; Singh H.; Yadav A. K.; Santos H. L. S.; Chundak M.; Abdelsalam I.; Heczko V. J.; Sitta E.; Ritala M.; Huo W.; Slater T. J. A.; Haigh S. J.; Camargo P. H. C. Ultralow Catalytic Loading for Optimised Electrocatalytic Performance of AuPt Nanoparticles to Produce Hydrogen and Ammonia. Angew. Chem., Int. Ed. 2024, 63 (29), e202405459 10.1002/anie.202405459. 38711309

[ref21] da Silva K. N.; Shetty S.; Sullivan–Allsop S.; Cai R.; Wang S.; Quiroz J.; Chundak M.; dos Santos H. L. S.; Abdelsalam I.; Oropeza F. E.; de la Peña O’Shea V. A.; Heikkinen N.; Sitta E.; Alves T. V.; Ritala M.; Huo W.; Slater T. J. A.; Haigh S. J.; Camargo P. H. C. Au@AuPd Core-Alloyed Shell Nanoparticles for Enhanced Electrocatalytic Activity and Selectivity under Visible Light Excitation. ACS Nano 2024, 18 (35), 24391–24403. 10.1021/acsnano.4c07076. 39164202 PMC11386439

[ref22] Jiang X.; Qiu X.; Fu G.; Sun J.; Huang Z.; Sun D.; Xu L.; Zhou J.; Tang Y. Highly Simple and Rapid Synthesis of Ultrathin Gold Nanowires with (111)-Dominant Facets and Enhanced Electrocatalytic Properties. J. Mater. Chem. A 2018, 6 (36), 17682–17687. 10.1039/C8TA06676K.

[ref23] Xue Q.; Bai X. Y.; Zhao Y.; Li Y. N.; Wang T. J.; Sun H. Y.; Li F. M.; Chen P.; Jin P.; Yin S.-B.; Chen Y. Au Core-PtAu Alloy Shell Nanowires for Formic Acid Electrolysis. J. Energy Chem. 2022, 65, 94–102. 10.1016/j.jechem.2021.05.034.

[ref24] Lu Y.; Jia X.; Ma Z.; Li Y.; Yue S.; Liu X.; Zhang J. W5+–W5+ Pair Induced LSPR of W18O49 to Sensitize ZnIn2S4 for Full-Spectrum Solar-Light-Driven Photocatalytic Hydrogen Evolution. Adv. Funct. Mater. 2022, 32 (35), 2203638 10.1002/adfm.202203638.

[ref25] Duan M.; Hu C.; Li H.; Chen Y.; Chen R.; Gong W.; Lu Z.; Zhang N.; Long R.; Song L.; Xiong Y. Synergizing Inter and Intraband Transitions in Defective Tungsten Oxide for Efficient Photocatalytic Alcohol Dehydration to Alkenes. JACS Au 2022, 2 (5), 1160–1168. 10.1021/jacsau.2c00146. 35647591 PMC9131368

[ref26] Li W. X.; Liu Z. Y.; Yang S. C.; Wu J. N.; Sun L.; Ma E. G.; Yang H. G.; Guo X. Highly Dispersed Pt Species Anchored on W18O49 Nanowires Mediate Efficient and Durable Hydrogen Evolution in Acidic Water. Sci. China Mater. 2022, 65 (12), 3435–3441. 10.1007/s40843-022-2258-3.

[ref27] Choi C. H.; Kim M.; Kwon H. C.; Cho S. J.; Yun S.; Kim H.-T.; Mayrhofer K. J. J.; Kim H.; Choi M. Tuning Selectivity of Electrochemical Reactions by Atomically Dispersed Platinum Catalyst. Nat. Commun. 2016, 7 (1), 10922 10.1038/ncomms10922. 26952517 PMC4786782

[ref28] Wan R.; Luo M.; Wen J.; Liu S.; Kang X.; Tian Y. Pt-Co Single Atom Alloy Catalysts: Accelerated Water Dissociation and Hydrogen Evolution by Strain Regulation. J. Energy Chem. 2022, 69, 44–53. 10.1016/j.jechem.2021.12.045.

[ref29] Dupin J. C.; Gonbeau D.; Vinatier P.; Levasseur A. Systematic XPS Studies of Metal Oxides, Hydroxides and Peroxides. Phys. Chem. Chem. Phys. 2000, 2 (6), 1319–1324. 10.1039/a908800h.

[ref30] Zhong X.; Sun Y.; Chen X.; Zhuang G.; Li X.; Wang J. G. Mo Doping Induced More Active Sites in Urchin-Like W18O49 Nanostructure with Remarkably Enhanced Performance for Hydrogen Evolution Reaction. Adv. Funct. Mater. 2016, 26 (32), 5778–5786. 10.1002/adfm.201601732.

[ref31] Kumar A.; Choudhary P.; Kumar A.; Camargo P. H. C.; Krishnan V. Recent Advances in Plasmonic Photocatalysis Based on TiO2 and Noble Metal Nanoparticles for Energy Conversion, Environmental Remediation, and Organic Synthesis. Small 2022, 18 (1), 2101638 10.1002/smll.202101638. 34396695

[ref32] Quiroz J.; Barbosa E. C. M.; Araujo T. P.; Fiorio J. L.; Wang Y.-C.; Zou Y.-C.; Mou T.; Alves T. V.; de Oliveira D. C.; Wang B.; Haigh S. J.; Rossi L. M.; Camargo P. H. C. Controlling Reaction Selectivity over Hybrid Plasmonic Nanocatalysts. Nano Lett. 2018, 18 (11), 7289–7297. 10.1021/acs.nanolett.8b03499. 30352162 PMC6348440

[ref33] Bezerra L. S.; Belhout S. A.; Wang S.; Quiroz J.; de Oliveira P. F. M.; Shetty S.; Rocha G.; Santos H. L. S.; Frindy S.; Oropeza F. E.; de la Peña O’Shea V. A.; Kallio A.-J.; Huotari S.; Huo W.; Camargo P. H. C. Triple Play of Band Gap, Interband, and Plasmonic Excitations for Enhanced Catalytic Activity in Pd/HxMoO3 Nanoparticles in the Visible Region. ACS Appl. Mater. Interfaces 2024, 16 (9), 11467–11478. 10.1021/acsami.3c17101. 38382920 PMC11393804

[ref34] Choi C. H.; Kim M.; Kwon H. C.; Cho S. J.; Yun S.; Kim H. T.; Mayrhofer K. J. J.; Kim H.; Choi M. Tuning Selectivity of Electrochemical Reactions by Atomically Dispersed Platinum Catalyst. Nat. Commun. 2016, 7 (1), 1–9. 10.1038/ncomms10922. PMC4786782 26952517

[ref35] Parthasarathy P.; Virkar A. V. Electrochemical Ostwald Ripening of Pt and Ag Catalysts Supported on Carbon. J. Power Sources 2013, 234, 82–90. 10.1016/j.jpowsour.2013.01.115.

[ref36] Gommes C. J. Ostwald Ripening of Confined Nanoparticles: Chemomechanical Coupling in Nanopores. Nanoscale 2019, 11 (15), 7386–7393. 10.1039/C9NR01349K. 30938749

[ref37] Bai H.; Lam S. H.; Yang J.; Cheng X.; Li S.; Jiang R.; Shao L.; Wang J. A Schottky-Barrier-Free Plasmonic Semiconductor Photocatalyst for Nitrogen Fixation in a “One-Stone-Two-Birds” Manner. Adv. Mater. 2022, 34 (2), 2104226 10.1002/adma.202104226. 34655458

[ref38] Hu J.; Guo T.; Zhong X.; Li J.; Mei Y.; Zhang C.; Feng Y.; Sun M.; Meng L.; Wang Z.; Huang B.; Zhang L.; Wang Z. In Situ Reconstruction of High-Entropy Heterostructure Catalysts for Stable Oxygen Evolution Electrocatalysis under Industrial Conditions. Adv. Mater. 2024, 36 (14), 2310918 10.1002/adma.202310918. 38170168

[ref39] Clark S. J.; Segall M. D.; Pickard C. J.; Hasnip P. J.; Probert M. I. J.; Refson K.; Payne M. C. First principles methods using CASTEP. Z. Kristallogr. Cryst. Mater. 2005, 220 (5–6), 567–570. 10.1524/zkri.220.5.567.65075.

[ref40] Ernzerhof M.; Scuseria G. E. Assessment of the Perdew–Burke–Ernzerhof Exchange-Correlation Functional. J. Chem. Phys. 1999, 110 (11), 5029–5036. 10.1063/1.478401. 15268348

[ref41] Fan X.; Liu C.; Gao B.; Li H.; Zhang Y.; Zhang H.; Gao Q.; Cao X.; Tang Y. Electronic Structure Engineering of Pt Species over Pt/WO3 toward Highly Efficient Electrocatalytic Hydrogen Evolution. Small 2023, 19 (32), 2301178 10.1002/smll.202301178. 37066750

