# Supplementary material for: Earth-Abundant W18O49 Coupled with Minimal Pt for Enhanced Hydrogen Evolution under Dark and Visible Light Conditions
Source: ACS Appl Mater Interfaces. 2025 Mar 11;17(11):16909–19. doi: 10.1021/acsami.4c22952 (PMC11931485; doi:10.1021/acsami.4c22952)
Supplement: Supplementary file 1 — am4c22952_si_001.pdf [file am4c22952_si_001.pdf]

Supporting Information for

Earth-abundant $W_{18}O_{49}$ Coupled with Minimal Pt for Enhanced Hydrogen Evolution under Dark and Visible Light Conditions

Hugo L. S. Santos,[1†] Md Mofakkharulhashan,[1†] Shiqi Wang,[1] Eric. V. Formo,[2] Mykhailo Chundak,[1] Mikko Ritala,[1] Wenyi Huo,[3,4*] and Pedro H. C. Camargo[1*]

[1] Department of Chemistry, University of Helsinki, A.I. Virtasen aukio 1, PO Box 55, FIN–00560 Helsinki, Finland

[2] Georgia Electron Microscopy, University of Georgia, Athens, GA, 30602 USA

[3] College of Mechanical and Electrical Engineering, Nanjing Forestry University. Nanjing, 210037, P. R. China.

[4] NOMATEN Centre of Excellence, National Centre for Nuclear Research. Otwock, 05-400, Poland

[†]These authors contributed equally to this work

** Corresponding authors. Email: pedro.camargo@helsinki.fi, wenyi.huo@ncbj.gov.pl*

Table S1. Pt concentration of the Pt-$W_{18}O_{49}$ samples measured by MS-AES. The quantity of Pt detected was close to the quantity added in the synthesis, indicating accurate control of the Pt loading.

	% wt. of Pt
0.4Pt-$W_{18}O_{49}$	0.412±0.013
0.8Pt-$W_{18}O_{49}$	0.832±0.013
1.6Pt-$W_{18}O_{49}$	1.629±0.031

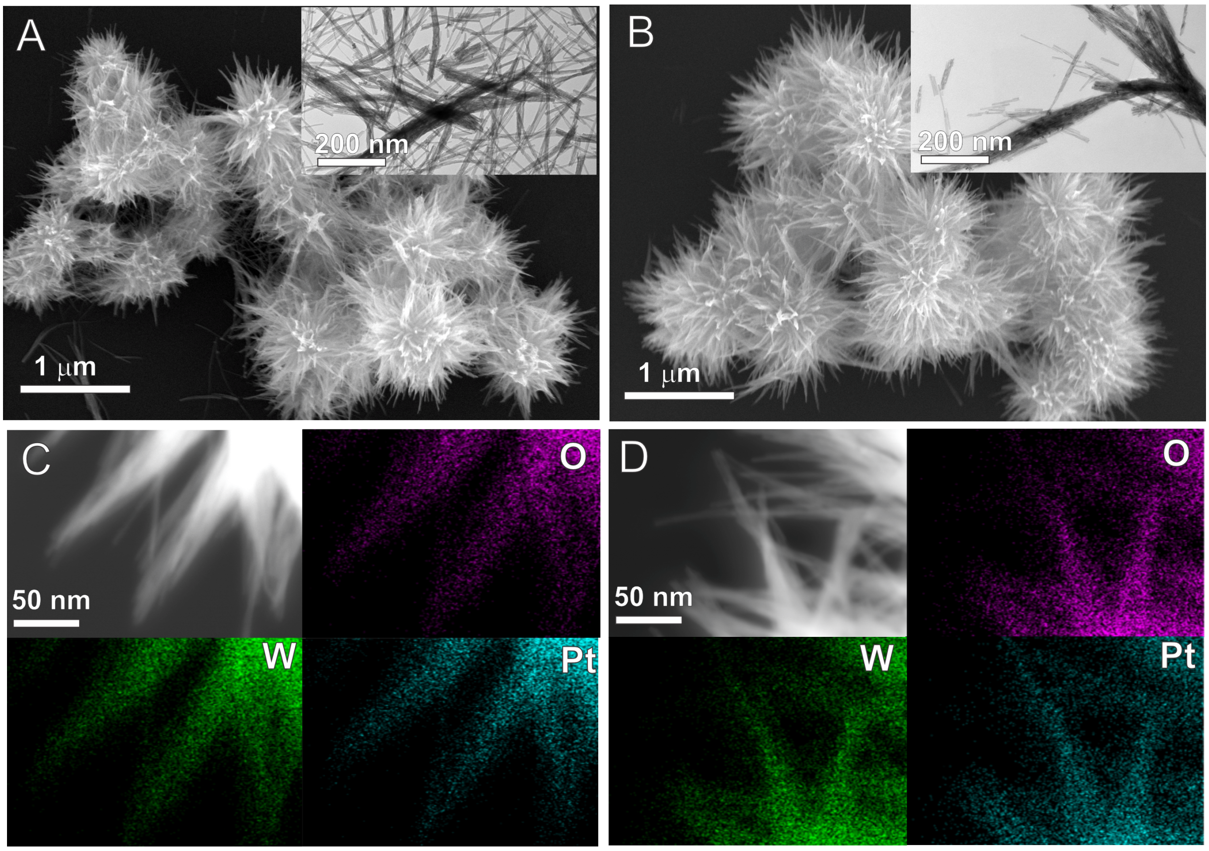

Figure S1. **Structural and compositional characterization of 0.8Pt-$W_{18}O_{49}$ and 0.4Pt-$W_{18}O_{49}$ nanourchins.** SEM and TEM images depicting the nanoflower morphology of (A) 0.4Pt-$W_{18}O_{49}$ and (B) 0.8Pt-$W_{18}O_{49}$. The inserted TEM images show the nanowires containing Pt clusters. STEM-HAADF and corresponding STEM-EDS maps for the distribution of O (pink), W (green) and Pt (blue) for (C) 0.4Pt-$W_{18}O_{49}$ and (D) 0.8Pt-$W_{18}O_{49}$, confirming the uniform Pt incorporation.

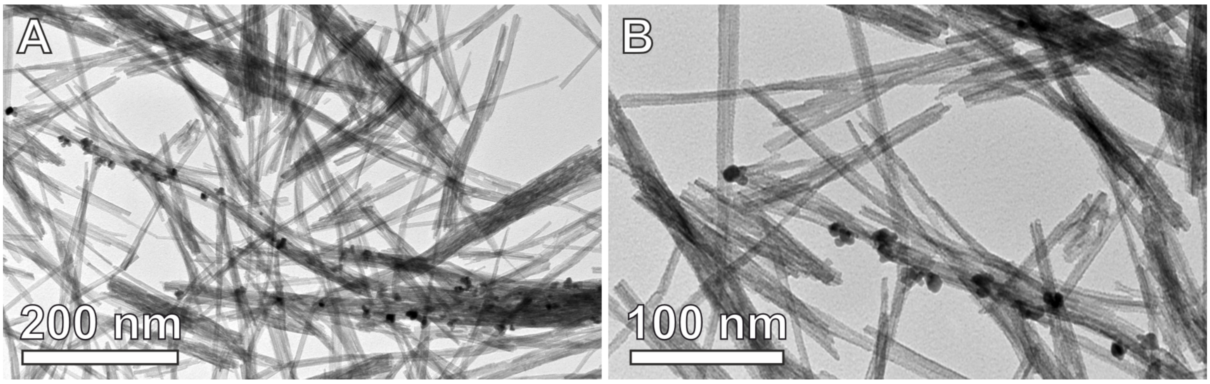

Figure S2. **Morphological characterization of the 1.6Pt-$W_{18}O_{49}$ nanourchins.** (A) Low and (B) high magnification bright-field TEM images depicting the nanourchins of 1.6Pt-$W_{18}O_{49}$ containing Pt clusters attached on the surface. The irregular Pt clusters had a length size of 12 nm.

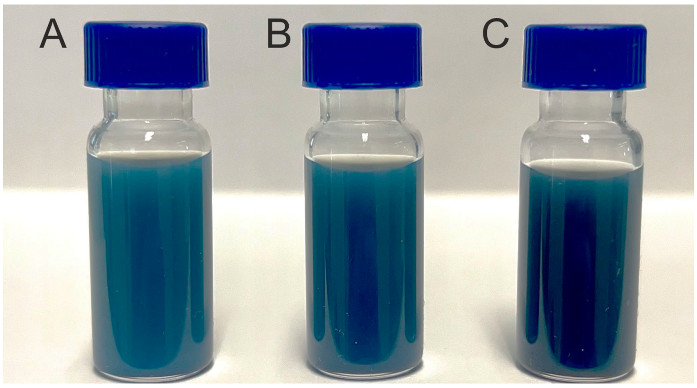

Figure S3. **Optical Appearance of Pt- $W_{18}O_{49}$ dispersions.** Solution dispersions of 0.4Pt-$W_{18}O_{49}$ (A), 0.8Pt-$W_{18}O_{49}$ (B), and 1.6Pt-$W_{18}O_{49}$ (C) were prepared. The image demonstrates the correlation between increasing Pt concentration and solution color intensification.

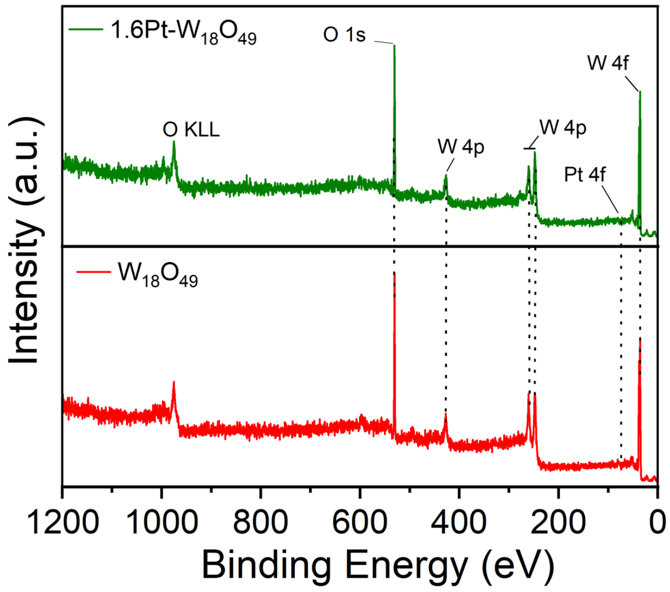

Figure S4. XPS survey spectra. XPS survey spectra of $W_{18}O_{49}$ and 1.6Pt-$W_{18}O_{49}$ showing the presence of W and O. No Pt peaks were detected in the 1.6Pt-$W_{18}O_{49}$ survey spectrum, likely due to low Pt concentration and XPS sensitivity limitations.

Table S2. XPS parameters for W 4f spectra of $W_{18}O_{49}$ and 1.6Pt-$W_{18}O_{49}$ samples.

Sample	Species	B.E. (eV)		Area		%$_{Area}$
		$4f_{7/2}$	$4f_{5/2}$	$4f_{7/2}$	$4f_{5/2}$	
$W_{18}O_{49}$	W^{5+}	34.6	36.7	17700.1	32946.4	12
	W^{6+}	35.9	38.1	202763.5	171235.8	88
1.6Pt-$W_{18}O_{49}$	W^{6+}	35.7	37.9	232329.8	171550.5	-

Table S3. XPS parameters for O 1s spectra of $W_{18}O_{49}$ and 1.6Pt-$W_{18}O_{49}$ samples.

Sample	Species	B.E. (eV)	Area	%$_{Area}$
$W_{18}O_{49}$	O^{2-}	530.6	168327.2	54
	O-H	531.1	144733.3	46
1.6Pt-$W_{18}O_{49}$	O^{2-}	530.5	150966.7	51
	O-H	531.0	142216.1	49

Table S4. Crystallite size for $W_{18}O_{49}$ and Pt-$W_{18}O_{49}$ samples calculated by Scherrer equation.

Samples	Crystalline size (nm)
$W_{18}O_{49}$	11.66
0.4Pt-$W_{18}O_{49}$	13.64
0.8Pt-$W_{18}O_{49}$	14.34
1.6Pt-$W_{18}O_{49}$	14.81

The Scherrer equation is $D = \frac{K\lambda}{\beta\ cos\theta}$, where D is the crystalline size, K is the shape factor, λ is the X-ray wavelength, β is the half the maximum intensity (FWHM) and θ is the Bragg angle.

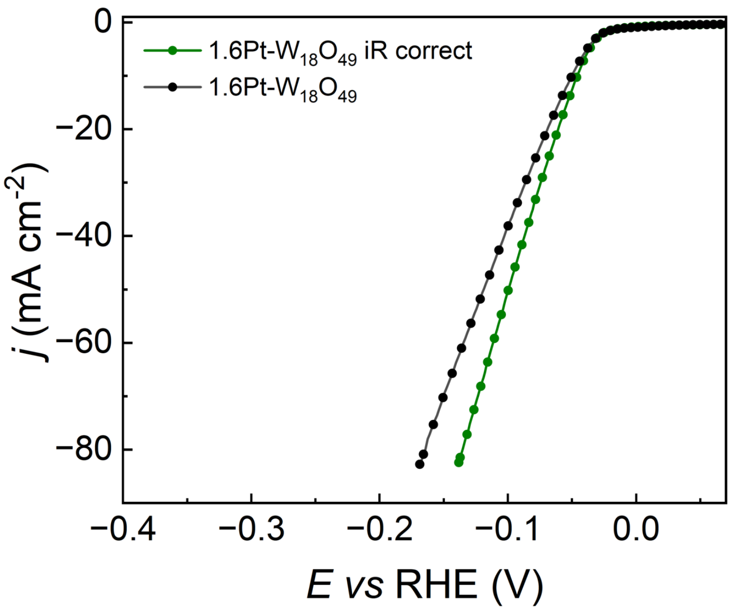

Figure S5. **Electrochemical performance of 1.6Pt-$W_{18}O_{49}$ under dark conditions**. Linear sweep voltammograms (LSVs) of the 1.6Pt-$W_{18}O_{49}$ electrocatalyst were recorded in 0.5 M H_2SO_4 solution under dark conditions, both with and without iR compensation. A minor current shift was observed because of *iR* correction.

Table S5. HER performance comparison of the state-of-the-art noble and non-noble metal based electrocatalysts in acid electrolytes.

Electrode materials	$\eta_{10\ mA\ cm^{-2}}$ (mV)	Tafel (mV dec^{-1})	Stability (h)	Electrolyte	References
**1.6Pt-$W_{18}O_{49}$**	**45**	**30**	**24**	**0.5 M H_2SO_4**	**our work**
Ru@C_2N	22	30	-	0.5 M H_2SO_4	1
NbPt/$W_{18}O_{49}$ NSs	37	23	18	0.5 M H_2SO_4	2
VS_2 nanosheets	43	36	60	0.5 M H_2SO_4	3
Ni@NC@MoS_2	82	47.5	-	0.5 M H_2SO_4	4
Mo-doped ReS_2 nanospheres	81	62	50	0.5 M H_2SO_4	5
Pt decorated SnS_2	117	69	12	0.5 M H_2SO_4	6
Pd doped $W_{18}O_{49}$ NFs	137	35	65	0.5 M H_2SO_4	7
Pt-SA/α-MoO_x	52	123	20	0.5 M H_2SO_4	8
Mn-Doped FeP/$Co_3(PO_4)_2$	27	44	8	0.5 M H_2SO_4	9
Pt-Ru dimers	35	28.9	10	0.5 M H_2SO_4	10
Pd/Cu-Pt NRs	22.8	25	15	0.5 M H_2SO_4	11
$Mo_2TiC_2T_x$–Pt_{SA}	30	30	100	0.5 M H_2SO_4	12
Pt_1/NMHCS	40	56	-	0.5 M H_2SO_4	13
Ni@NC@MoS_2	82	47.5	8	0.5 M H_2SO_4	4
MoO_2/$MoSe_2$	181	49.1	8	0.5 M H_2SO_4	14
Pd ND/DR-MoS_2	120	41	2.7	0.5 M H_2SO_4	15
α-INS nanosheets	105	40	40	0.5 M H_2SO_4	16
1T-WS_2	151	70	4	0.5 M H_2SO_4	17
Se-enriched $NiSe_2$ nanosheet	117	32	67	0.5 M H_2SO_4	18
Mo doped $W_{18}O_{49}$ NFs	262	49	24	0.5 M H_2SO_4	19
10% Mo-$W_{18}O_{49}$	45	54	12	0.5 M H_2SO_4	20
PtNi/CNF	34	31	10	0.5 M H_2SO_4	21
$IrNi_{0.58}Fe_{0.82}$	24	34.6	5.5	0.5 M $HClO_4$	22
Ni/Ru-doped Pt @Ni/Pt-doped	29.6	35	-	0.1 M $HClO_4$	23

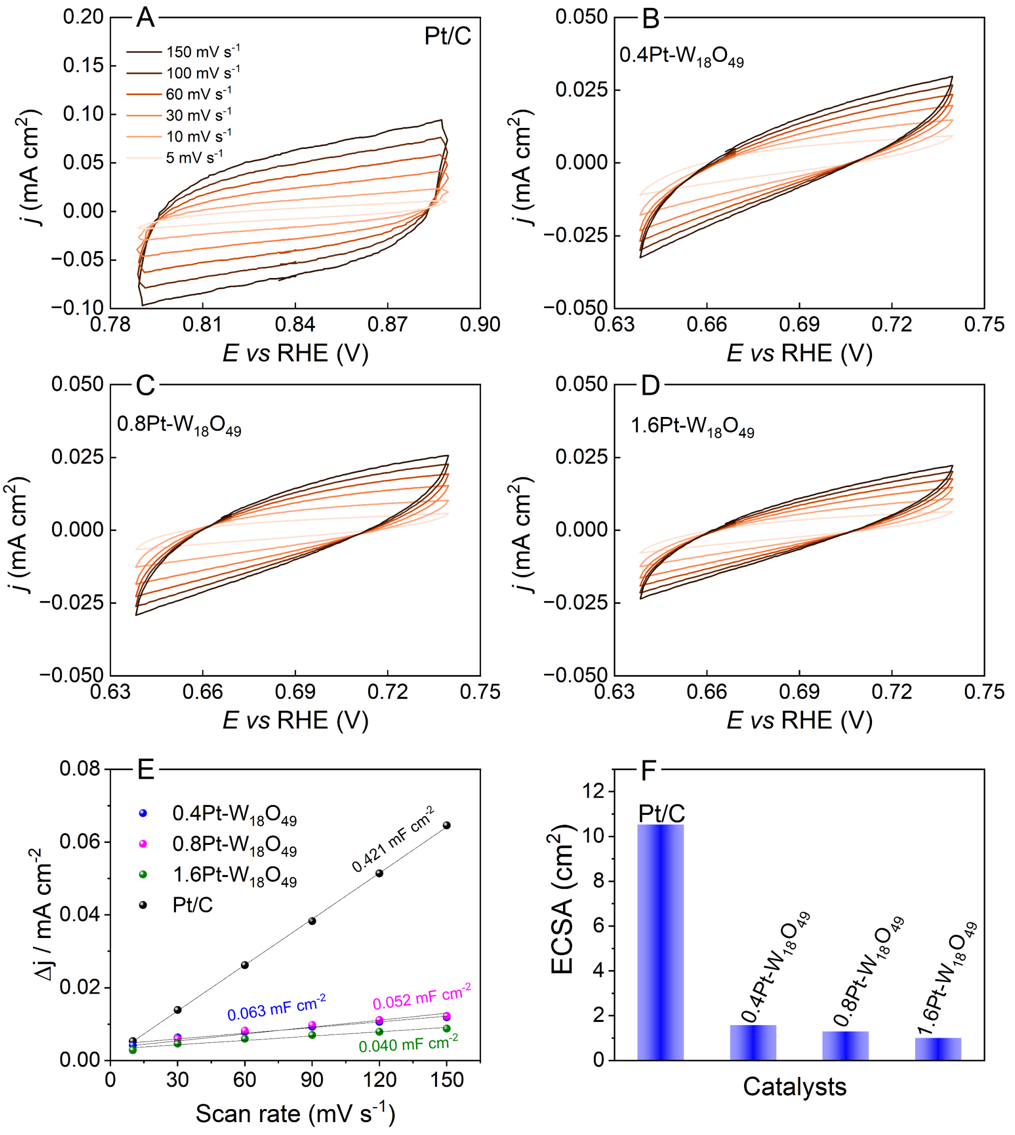

Figure S6. Electrochemical surface area (ECSA) analysis of Pt/C and Pt-$W_{18}O_{49}$ samples. Voltammograms measured in a non-faradaic region at different scan rates (10, 30, 60, 90, 120 and 150 mV s^{-1}) for (A) 20 wt% Pt/C, (B) 0.4Pt-$W_{18}O_{49}$, (C) 0.8Pt-$W_{18}O_{49}$ and (D) 1.6Pt-$W_{18}O_{49}$ in 0.5 mol L^{-1} H_2SO_4. (E) Δj vs Scan rate plot for Pt/C and Pt-$W_{18}O_{49}$ samples. (F) ECSA values for Pt/C and Pt-$W_{18}O_{49}$ samples. The Δj was determined using $(j_{anode}-j_{cathode})/2$ at 0.69 V for Pt-$W_{18}O_{49}$ samples and at 0.84 V for Pt/C. (F). The ECSA was determined using $ECSA = \frac{C_{dl}}{C_s}$, where C_s is the specific capacitance for a flat material. In our calculation, the C_s was considered 40 µF cm^{-2}.[24]

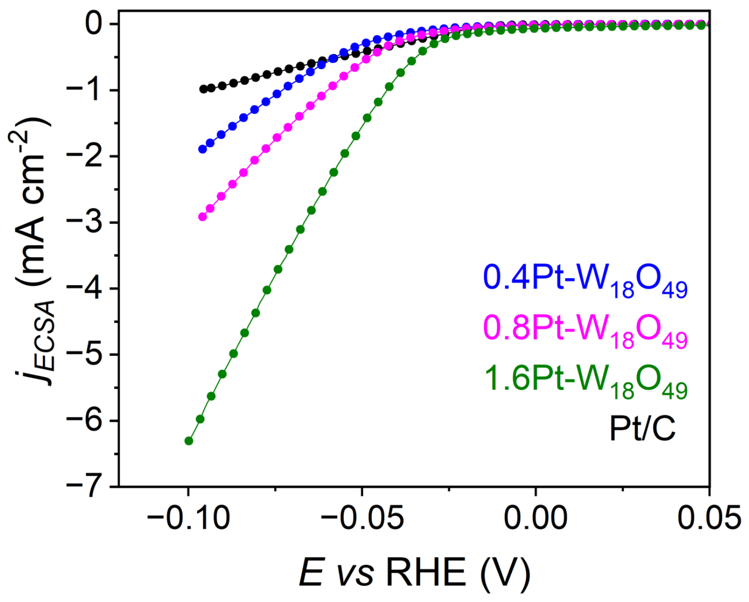

Figure S7. **Electrocatalytic activity normalized by ECSA**. Linear voltammograms with current normalized by the ECSA area. The LSVs normalized by ECSA demonstrate that the activity of the Pt-$W_{18}O_{49}$ samples is not only correlated to the electroactive area but a synergic effect between $W_{18}O_{49}$ and Pt.

Table S6. EIS parameters, obtained by fitting a Randles circuit for $W_{18}O_{49}$ and Pt-$W_{18}O_{49}$. The lower charge transfer resistance (R_{ct}) values observed for LED-illuminated experiments and Pt-richer samples suggest enhanced electron transfer kinetics and improved HER performance.

Sample		R_s (Ω cm^2)	**R_{ct} (Ω cm^2)**	CPE	
				P	n
0.4Pt-$W_{18}O_{49}$	Dark	8.3	**113.1**	0.0085	0.81
	LED 740nm	8.4	**70.8**	0.0086	0.81
0.8Pt-$W_{18}O_{49}$	Dark	8.0	**41.6**	0.010	0.80
	LED 740nm	7.9	**23.5**	0.011	0.83
1.6Pt-$W_{18}O_{49}$	Dark	7.9	**10.5**	0.022	0.92
	LED 740nm	7.6	**9.2**	0.021	0.96

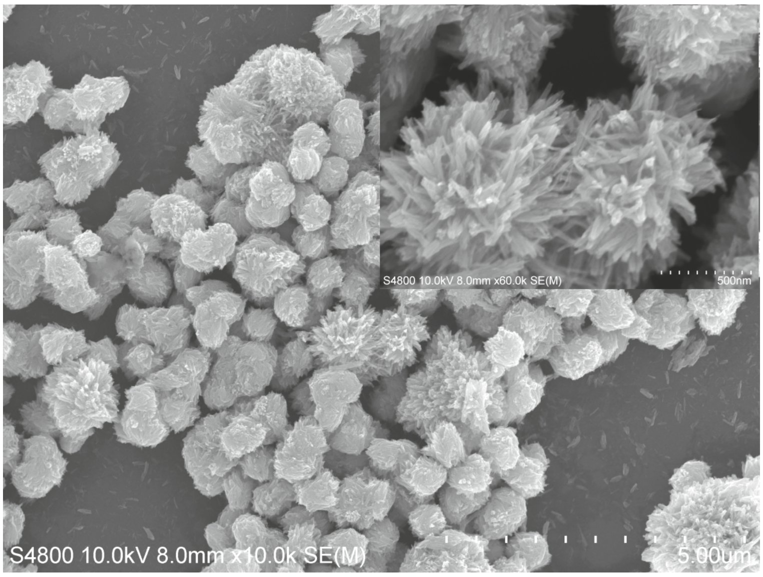

Figure S8. Morphological Stability of 1.6Pt-$W_{18}O_{49}$. SEM image of 1.6Pt-$W_{18}O_{49}$ after undergoing a 24-hour stability test. While the overall morphology remains relatively unchanged, some degradation is evident, particularly in the wire-like structures, which appear more fragmented.

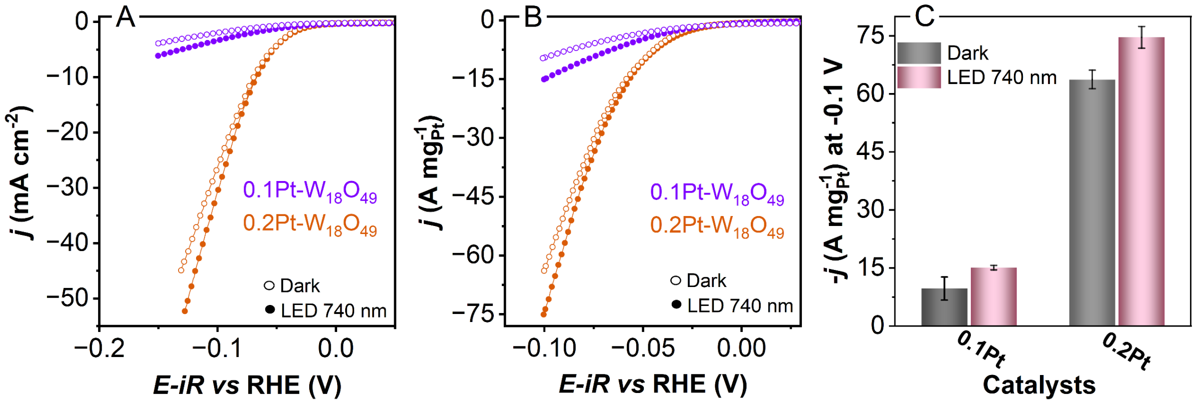

Figure S9. Electrocatalytic performance of 0.2Pt-$W_{18}O_{49}$ and 0.1Pt-$W_{18}O_{49}$ samples. (A) iR correct LSVs normalized by geometric area, (B) LSVs normalized by mass, (C) mass activity at -0.1 V_{RHE} for 0.2Pt-W18O49 and 0.1Pt-W18O49 samples. . All measurements were obtained in argon purged with 0.5 mol L-1 H2SO4 at 25 °C.

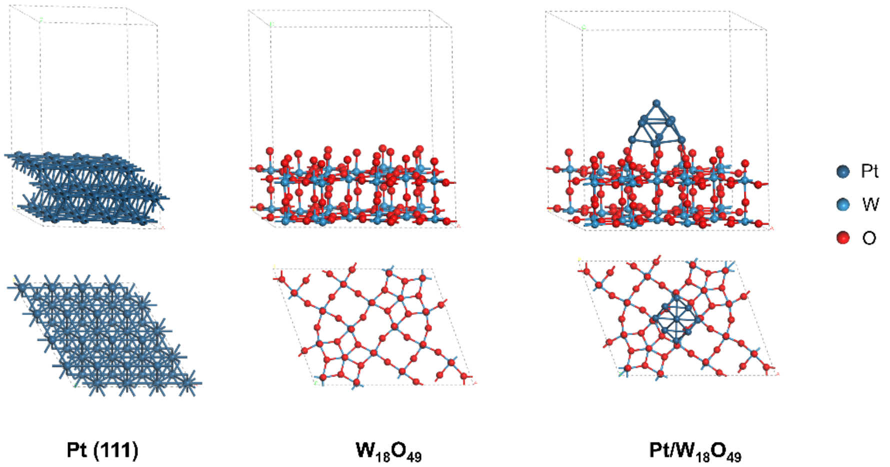

Figure S10. **Structural models for DFT**. Structural models for Pt (111), $W_{18}O_{49}$, and Pt-$W_{18}O_{49}$ employed in this work.

Reference

(1) Mahmood, J.; Li, F.; Jung, S. M.; Okyay, M. S.; Ahmad, I.; Kim, S. J.; Park, N.; Jeong, H. Y.; Baek, J. B. An Efficient and PH-Universal Ruthenium-Based Catalyst for the Hydrogen Evolution Reaction. *Nat Nanotechnol* **2017**, *12* (5), 441–446. https://doi.org/10.1038/NNANO.2016.304.

(2) Electronic Modulation of Novel W 18 O 49 Nanoshuttles for Efficient Hydrogen Evolution Reaction. *Nanotechnology* **2020,** *31,* 425705. https://doi.org/10.1088/1361-6528/ab9fb5.

(3) Zhang, J.; Zhang, C.; Wang, Z.; Zhu, J.; Wen, Z.; Zhao, X.; Zhang, X.; Xu, J.; Lu, Z. Synergistic Interlayer and Defect Engineering in VS2 Nanosheets toward Efficient Electrocatalytic Hydrogen Evolution Reaction. *Small* **2018**, *14* (9), 1703098. https://doi.org/10.1002/SMLL.201703098.

(4) Ali Shah, S.; Shen, X.; Xie, M.; Zhu, G.; Ji, Z.; Zhou, H.; Xu, K.; Yue, X.; Yuan, A.; Zhu, J.; Chen A Shah, Y. S.; Shen, X.; Zhu, G.; Ji, Z.; Zhou, H.; Xu, K.; Yue, X.; Zhu, J.; Chen, Y.; Xie, M.; Yuan, A. Nickel@Nitrogen-Doped Carbon@MoS2 Nanosheets: An Efficient Electrocatalyst for Hydrogen Evolution Reaction. *Small* **2019**, *15* (9), 1804545. https://doi.org/10.1002/SMLL.201804545.

(5) Xu, J.; Fang, C.; Zhu, Z.; Wang, J.; Yu, B.; Zhang, J. Nanoscale Engineering and Mo-Doping of 2D Ultrathin ReS2 Nanosheets for Remarkable Electrocatalytic Hydrogen Generation. *Nanoscale* **2020**, *12* (32), 17045–17052. https://doi.org/10.1039/D0NR03693E.

(6) Liu, G.; Qiu, Y.; Wang, Z.; Zhang, J.; Chen, X.; Dai, M.; Jia, D.; Zhou, Y.; Li, Z.; Hu, P. Efficiently Synergistic Hydrogen Evolution Realized by Trace Amount of Pt-Decorated Defect-Rich SnS2 Nanosheets. *ACS Appl Mater Interfaces* **2017**, *9* (43), 37750–37759. https://doi.org/10.1021/ACSAMI.7B11413

(7) Zhao, Y.; Tang, Q.; Yang, P.; He, B.; Li, R.; Chemcomm, /; Communication, C. Robust Electrocatalysts from Metal Doped W18O49 Nanofibers for Hydrogen Evolution. *Chemical Communications* **2017**, *53* (31), 4323–4326. https://doi.org/10.1039/C7CC01249G.

(8) Xu, J.; Zhang, C.; Liu, H.; Sun, J.; Xie, R.; Qiu, Y.; Lü, F.; Liu, Y.; Zhuo, L.; Liu, X.; Luo, J. Amorphous MoOX-Stabilized Single Platinum Atoms with Ultrahigh Mass Activity for Acidic Hydrogen Evolution. *Nano Energy* **2020**, *70*, 104529. https://doi.org/10.1016/J.NANOEN.2020.104529.

(9) Liu, H.; Peng, X.; Liu, X.; Qi, G.; Luo, J. Porous Mn-Doped FeP/Co 3 (PO 4) 2 Nanosheets as Efficient Electrocatalysts for Overall Water Splitting in a Wide PH Range. *ChemSusChem* **2019**, *12* (7), 1334–1341. https://doi.org/10.1002/CSSC.201802437.

(10) Zhang, L.; Si, R.; Liu, H.; Chen, N.; Wang, Q.; Adair, K.; Wang, Z.; Chen, J.; Song, Z.; Li, J.; Banis, M. N.; Li, R.; Sham, T. K.; Gu, M.; Liu, L. M.; Botton, G. A.; Sun, X. Atomic Layer Deposited Pt-Ru Dual-Metal Dimers and Identifying Their Active Sites for Hydrogen Evolution Reaction. *Nature Communications 2019 10:1* **2019**, *10* (1), 1–11. https://doi.org/10.1038/s41467-019-12887-y.

(11) Chao, T.; Luo, X.; Chen, W.; Jiang, B.; Ge, J.; Lin, Y.; Wu, G.; Wang, X.; Hu, Y.; Zhuang, Z.; Wu, Y.; Hong, X.; Li, Y. Atomically Dispersed Copper–Platinum Dual Sites Alloyed with Palladium Nanorings Catalyze the Hydrogen Evolution Reaction. *Angew Chem Int Ed* **2017**, *56* (50), 16047–16051. https://doi.org/10.1002/ANIE.201709803.

(12) Zhang, J.; Zhao, Y.; Guo, X.; Chen, C.; Dong, C. L.; Liu, R. S.; Han, C. P.; Li, Y.; Gogotsi, Y.; Wang, G. Single Platinum Atoms Immobilized on an MXene as an Efficient Catalyst for the Hydrogen Evolution Reaction. *Nature Catalysis 2018 1:12* **2018**, *1* (12), 985–992. https://doi.org/10.1038/s41929-018-0195-1.

(13) Kuang, P.; Wang, Y.; Zhu, B.; Xia, F.; Tung, C. W.; Wu, J.; Chen, H. M.; Yu, J. Pt Single Atoms Supported on N-Doped Mesoporous Hollow Carbon Spheres with Enhanced Electrocatalytic H2-Evolution Activity. *Advanced Materials* **2021**, *33* (18), 2008599. https://doi.org/10.1002/ADMA.202008599.

(14) Chen, X.; Liu, G.; Zheng, W.; Feng, W.; Cao, W.; Hu, W.; Hu, P. A. Vertical 2D MoO2/MoSe2 Core–Shell Nanosheet Arrays as High-Performance Electrocatalysts for Hydrogen Evolution Reaction. *Adv Funct Mater* **2016**, *26* (46), 8537–8544. https://doi.org/10.1002/ADFM.201603674.

(15) Qi, K.; Yu, S.; Wang, Q.; Zhang, W.; Fan, J.; Zheng, W.; Cui, X. Decoration of the Inert Basal Plane of Defect-Rich MoS2 with Pd Atoms for Achieving Pt-Similar HER Activity. *J Mater Chem A Mater* **2016**, *4* (11), 4025–4031. https://doi.org/10.1039/C5TA10337A.

(16) Long, X.; Li, G.; Wang, Z.; Zhu, H.; Zhang, T.; Xiao, S.; Guo, W.; Yang, S. Metallic Iron-Nickel Sulfide Ultrathin Nanosheets As a Highly Active Electrocatalyst for Hydrogen Evolution Reaction in Acidic Media. *J Am Chem Soc* **2015**, *137* (37), 11900–11903. https://doi.org/10.1021/JACS.5B07728.

(17) Lukowski, M. A.; Daniel, A. S.; English, C. R.; Meng, F.; Forticaux, A.; Hamers, R. J.; Jin, S. Highly Active Hydrogen Evolution Catalysis from Metallic WS2 Nanosheets. *Energy Environ Sci* **2014**, *7* (8), 2608–2613. https://doi.org/10.1039/C4EE01329H.

(18) Wang, F.; Li, Y.; Shifa, T. A.; Liu, K.; Wang, F.; Wang, Z.; Xu, P.; Wang, Q.; He, J. Selenium-Enriched Nickel Selenide Nanosheets as a Robust Electrocatalyst for Hydrogen Generation. *Angew Chem Int Ed* **2016**, *55* (24), 6919–6924. https://doi.org/10.1002/ANIE.201602802.

(19) Zhao, Y.; Tang, Q.; He, B.; Yang, P. Mo Incorporated W18O49 Nanofibers as Robust Electrocatalysts for High-Efficiency Hydrogen Evolution. *Int J Hydrogen Energy* **2017**, *42* (21), 14534–14546. https://doi.org/10.1016/J.IJHYDENE.2017.04.115.

(20) Zhong, X.; Sun, Y.; Chen, X.; Zhuang, G.; Li, X.; Wang, J. G. Mo Doping Induced More Active Sites in Urchin-Like W18O49 Nanostructure with Remarkably Enhanced Performance for Hydrogen Evolution Reaction. *Adv Funct Mater* **2016**, *26* (32), 5778–5786. https://doi.org/10.1002/ADFM.201601732.

(21) Chen, J.; Wang, J.; Chen, J.; Wang, L. A Bifunctional Electrocatalyst of PtNi Nanoparticles Immobilized on Three-Dimensional Carbon Nanofiber Mats for Efficient and Stable Water Splitting in Both Acid and Basic Media. *J Mater Sci* **2017**, *52* (22), 13064–13077. https://doi.org/10.1007/S10853-017-1410-1/TABLES/1.

(22) Fu, L.; Cheng, G.; Luo, W. Colloidal Synthesis of Monodisperse Trimetallic IrNiFe Nanoparticles as Highly Active Bifunctional Electrocatalysts for Acidic Overall Water Splitting. *J Mater Chem A Mater* **2017**, *5* (47), 24836–24841. https://doi.org/10.1039/C7TA08982A.

(23) Oh, A.; Young Kim, H.; Baik, H.; Kim, B.; Kaduba Chaudhari, N.; Hoon Joo, S.; Lee, K.; Oh, A.; Kim, B.; Chaudhari, N. K.; Lee, K.; Baik, H.; Kim, H. Y.; Joo, S. H. Topotactic Transformations in

an Icosahedral Nanocrystal to Form Efficient Water-Splitting Catalysts. *Advanced Materials* **2019**, *31* (1), 1805546. https://doi.org/10.1002/ADMA.201805546.

(24) Diao, J.; Qiu, Y.; Liu, S.; Wang, W.; Chen, K.; Li, H.; Yuan, W.; Qu, Y.; Guo, X. Interfacial Engineering of W2N/WC Heterostructures Derived from Solid-State Synthesis: A Highly Efficient Trifunctional Electrocatalyst for ORR, OER, and HER. *Advanced Materials* **2020**, *32* (7). 1905679. https://doi.org/10.1002/adma.201905679.
